# Genome-Wide and Phase-Specific DNA-Binding Rhythms of BMAL1 Control Circadian Output Functions in Mouse Liver

**DOI:** 10.1371/journal.pbio.1000595

**Published:** 2011-02-22

**Authors:** Guillaume Rey, François Cesbron, Jacques Rougemont, Hans Reinke, Michael Brunner, Felix Naef

**Affiliations:** 1 School of Life Sciences, Ecole Polytechnique Fédérale de Lausanne, Lausanne, Switzerland; 2 Swiss Institute of Bioinformatics, Bâtiment Génopode, Université de Lausanne, Lausanne, Switzerland; 3 Biochemistry Center, Universität Heidelberg, Heidelberg, Germany; 4 Department of Molecular Biology, University of Geneva, Geneva, Switzerland; 5 Medical Faculty, Institute of Clinical Chemistry and Laboratory Diagnostics, Universität Düsseldorf, Düsseldorf, Germany; 6 Leibniz Institute for Molecular Preventive Medicine, Universität Düsseldorf, Düsseldorf, Germany; Charité Universitätsmedizin Berlin, Germany

## Abstract

The mammalian circadian clock uses interlocked negative feedback loops in which the heterodimeric basic helix-loop-helix transcription factor BMAL1/CLOCK is a master regulator. While there is prominent control of liver functions by the circadian clock, the detailed links between circadian regulators and downstream targets are poorly known. Using chromatin immunoprecipitation combined with deep sequencing we obtained a time-resolved and genome-wide map of BMAL1 binding in mouse liver, which allowed us to identify over 2,000 binding sites, with peak binding narrowly centered around Zeitgeber time 6. Annotation of BMAL1 targets confirms carbohydrate and lipid metabolism as the major output of the circadian clock in mouse liver. Moreover, transcription regulators are largely overrepresented, several of which also exhibit circadian activity. Genes of the core circadian oscillator stand out as strongly bound, often at promoter and distal sites. Genomic sequence analysis of the sites identified E-boxes and tandem E1-E2 consensus elements. Electromobility shift assays showed that E1-E2 sites are bound by a dimer of BMAL1/CLOCK heterodimers with a spacing-dependent cooperative interaction, a finding that was further validated in transactivation assays. BMAL1 target genes showed cyclic mRNA expression profiles with a phase distribution centered at Zeitgeber time 10. Importantly, sites with E1-E2 elements showed tighter phases both in binding and mRNA accumulation. Finally, analyzing the temporal profiles of BMAL1 binding, precursor mRNA and mature mRNA levels showed how transcriptional and post-transcriptional regulation contribute differentially to circadian expression phase. Together, our analysis of a dynamic protein-DNA interactome uncovered how genes of the core circadian oscillator crosstalk and drive phase-specific circadian output programs in a complex tissue.

## Abstract 2

Temporal mapping during a circadian day of binding sites for the BMAL1 transcription factor in mouse liver reveals genome-wide daily rhythms in DNA binding and uncovers output functions that are controlled by the circadian oscillator.

## Author Summary

The circadian clock is a timing system that allows organisms to keep behavioral, physiological, and cellular rhythms in resonance with daily environmental cycles. In mammals, such clocks use transcriptional regulatory loops in which the heterodimeric transcription factor BMAL1/CLOCK plays a central role. While defects in circadian clock function have been associated with diabetes, obesity, and cancer, the molecular links between the circadian clock and such output pathways are poorly characterized. Here, we mapped DNA-binding sites of BMAL1 in mouse liver during one circadian cycle. Our temporal analysis revealed widespread daily rhythms in DNA binding, with maximum levels peaking at midday. In the list of candidates, core circadian genes stood out as the most strongly bound, often showing multiple binding sites. Interestingly, BMAL1 targets were highly enriched in genes involved in carbohydrate and lipid metabolism, and also in transcription factors, in particular nuclear receptors. Our results suggest that the mammalian clock uses BMAL1 to control transcriptional output programs both directly and indirectly. Additionally, the DNA specificity of BMAL1 binding revealed the importance of tandem E-box elements, which may favor strong binding and precise timing of daily gene expression. Taken together, our work confirms BMAL1's primary function as a master regulator of the core circadian oscillator, while demonstrating that it also contributes in a more distributed fashion to a variety of output programs.

## Introduction

Circadian clocks provide higher organisms with cell-autonomous and organ-based metronomes that control temporally gated and tissue-specific gene expression or metabolic programs [1]–[4]. In the liver, such programs have been implicated in detoxification [5], glucose homeostasis [6],[7], cholesterol biosynthesis [8],[9], and gating of the cell cycle [10],[11]. The mammalian clock depends on a cell-autonomous [11] core oscillator that is built around interlocked transcriptional feedback loops. These use a variety of transcriptional regulators: the basic helix-loop-helix (bHLH) PAS domain proteins CLOCK, NPAS2, and BMAL1 [12],[13], orphan nuclear receptors of the REV-ERB [14] and ROR families [15], and the DEC bHLH repressors [16]. In addition, important co-regulators such as PER and CRY proteins mediate negative feedback by repressing their own transcriptional activators, BMAL1/CLOCK [17]–[20]. Among all these regulators, the *Bmal1* gene is the only single gene in the circadian network whose knockout results in arrhythmicity [21],[22]. BMAL1 functions as a heterodimeric complex, BMAL1/CLOCK, that activates transcription of its targets via E-boxes [12],[23],[24]. The DNA-binding activity of BMAL1/CLOCK is thought to cycle because of circadian changes in post-translational modifications [25],[26]. The core oscillator exerts its function by controlling temporally gated outputs, notably metabolic functions [5],[7],[27]. Transcriptional regulation of circadian output is known to occur both directly via the core clock transcription factors and indirectly, as, for example, via the PAR-bZip regulators DBP/TEF/HLF, which are themselves controlled by BMAL1/CLOCK [28]. Thus, circadian output function is controlled via a hierarchical network of transcription regulators that drives vast programs of tissue-specific gene expression both in the suprachiasmatic nucleus [29] and in peripheral tissues [29]–[34] in the mouse. Notably, these transcript rhythms cover the full range of expression phases, which thus begs the question about the mechanism behind phase-specific circadian gene expression. It has been proposed that virtually any peak expression phase can be achieved by suitably tuned regulatory sequences that integrate a small number of phase-specific core regulators [35]. Here we investigate the degree to which BMAL1 recruitment to the genomic DNA is itself rhythmic and to what extent peak binding carries phase information for downstream circadian mRNA expression.

To address these questions and further dissect the hierarchical structure of circadian clock networks, we perform a time series chromatin immunoprecipitation (ChIP) analysis for the master clock regulator BMAL1 in mouse liver. This allows us to identify a comprehensive set of direct BMAL1 targets in a circadianly controlled tissue, to model the DNA-binding specificity of BMAL1 in vivo, and to determine how tightly the phase of mRNA output follows rhythmic protein-DNA interactions. Our results reveal the pervasiveness of circadian protein-DNA interactions in a mammalian tissue by showing widespread rhythmic and phase-specific binding of BMAL1 to coding and non-coding genes. This enables us to characterize the cooperative interactions of BMAL1/CLOCK complexes at tandem E-box elements (E1-E2), and to emphasize the complexity of circadian phase control that involves transcriptional and post-transcriptional mechanisms.

## Results

### BMAL1 Binds Rhythmically to Thousands of Genomic Regions in Mouse Liver

**Figure 1:**
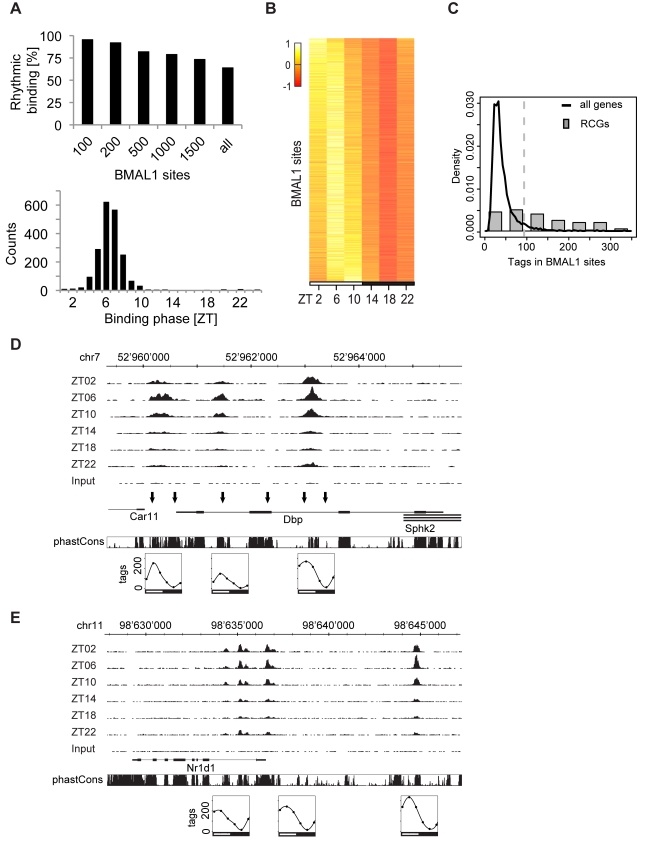
Time-resolved BMAL1 ChIP-Seq in mouse liver. (A) Top panel: Fraction of rhythmic BMAL1 sites in different subgroups. Sites were ranked according to binding strength using total number of tags over all the time points. Subgroups include all sites up to the indicated ranking. Lower panel: Histogram of binding phases peaks between ZT4 and ZT8. (B) Temporal profiles of BMAL1 binding ordered by phase. Only rhythmic profiles are plotted (Fisher test, *p*<0.05; see Materials and Methods). (C) Histogram of number of tags in BMAL1 binding sites for all sites (black curve) and a group of RCGs (grey bars), 63% of which (26 out of 41) are above the 95% quantile shown by the vertical dashed line. The RCGs include *Per1*/*2*/*3*, *Cry1*/*2*, *Dec1*/*2*, Rev-Erbα/β, *Ror*α/γ, *E4bp4,* and *Hlf*/*Tef*/*Dbp,* and show 41 binding sites all together. (D) BMAL1 ChIP-Seq data at the *Dbp* locus (visualized in the UCSC Genome Browser) show three rhythmic binding sites located at the promoter, in the first intron, and in the second intron. Notably, these overlap with DNase I hypersensitive sites [28], shown by the black arrows. The panels below show quantifications of BMAL1 binding. The scale is in number of non-redundant tags per 10 million mapped tags (see Materials and Methods). The PhastCons conservation score measures phylogenetic conservation among 20 placental mammals [85]. (E) UCSC Genome Browser view of BMAL1 ChIP-Seq data at the *Rev-Erb*α locus, showing two circadian binding sites close to its promoter and one upstream (−8 kb) site.

To obtain a time-resolved and genome-wide map of BMAL1 target sites, we performed ChIP in mouse liver at 4-h time intervals during one light-dark cycle. Following initial testing of ChIP efficiency by quantitative PCR (qPCR) (Figure S1), two independent BMAL1 ChIP time courses were subjected to ultra-high-throughput sequencing to yield about 20 million tags per time point (Table S1) and were analyzed via a bioinformatics pipeline that combines existing and novel methods. Briefly, we used the MACS software [36] to detect regions with enriched BMAL1 binding compared to an input chromatin sample (see Materials and Methods). To efficiently reject spurious signals and accurately estimate the location of binding sites, we developed a model-based deconvolution method for ChIP combined with deep sequencing (ChIP-Seq) data (see Text S1). We identified 2,049 bona fide BMAL1 binding sites in mouse liver. Among the top 200 sites, more than 90% are significantly rhythmic (Fisher test, *p*<0.05; see Materials and Methods), while the proportion drops to 60% for all sites (1,319 sites) (Figure 1A). Consistent with previously published results [24],[37], the binding phases are sharply distributed around Zeitgeber time (ZT) 4 to ZT8 (Figure 1A and 1B), which confirms BMAL1 as a highly phase-specific circadian transcription factor. At peak time, the binding signal (measured in number of unique tags in a site) spans over one order of magnitude, and sites near reference clock genes (RCGs) clearly stand out as the most strongly bound sites (Figure 1C; Text S3), i.e., 26 out of the 41 RCG sites are among the top 5% binding sites. In addition, RCGs often have multiple robustly rhythmic binding sites. For example, the *Dbp* gene has three sites: at the promoter and in the first and second introns (Figure 1D), with peak-to-trough amplitudes greater than 10-fold, similar to those measured with qPCR (Figure S1), with some residual binding at ZT18 compared to input chromatin. The three sites clearly overlap with DNase I hypersensitive sites mapped in [28] and also with evolutionarily conserved regions in the genome, suggesting that these sites are under purifying selection. Similarly, *Rev-Erb*α shows three strongly rhythmic sites, two near the promoter and one 8 kb upstream (Figure 1E), which could be involved in DNA looping with the promoter sites. A vast majority of RCGs, including the *Per1*/*2*, *Cry1*/*2*, *Dec1*/*2*, *Rev-Erb*β, *Ror*γ, *E4bp4,* and *Hlf*/*Tef* genes, show similarly strong signals (Figure S2). Moreover, we also find binding sites at recently identified targets like *Gys2* [38], *Nampt* [39],[40], and *Wee1* [10].

### BMAL1 Binding Sites Are Enriched in Promoter Regions and Are Evolutionarily Conserved

**Figure 2:**
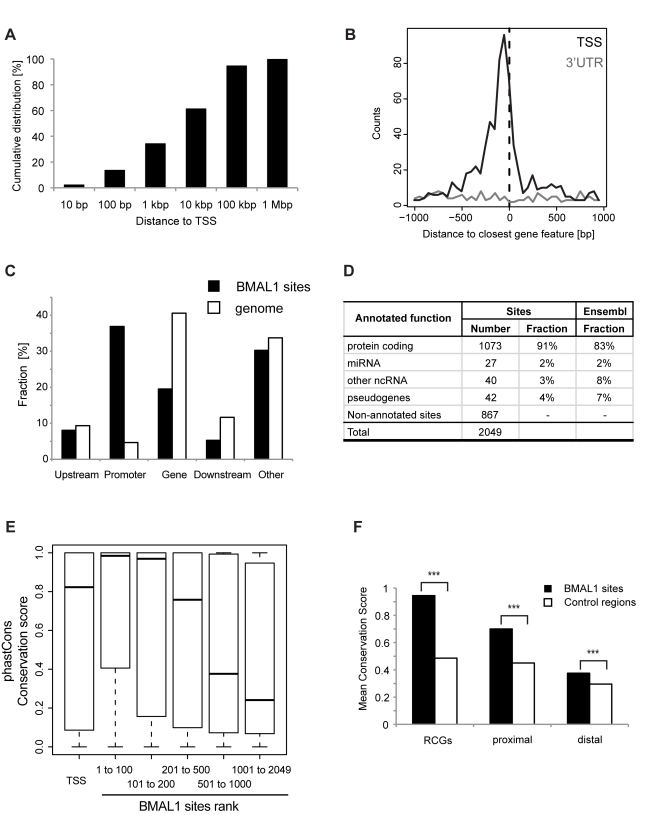
Genomic location and conservation of BMAL1 sites. (A) Cumulative distribution of BMAL1 site positions relative to the closest annotated Ensembl TSSs show that 40% of all sites are within 1 kb of a TSS. (B) Histogram of BMAL1 site positions, showing that BMAL1 sites cluster near TSSs, with a maximal density near −100 bp. No clustering is found near gene 3′ ends. (C) Positioning of BMAL1 sites near coding genes. Sites were assigned according to the following definitions: promoter (site is within ±2 kb of an annotated TSS), upstream (−10 kb to −2 kb), gene (+2 kb to the polyadenylation site), downstream (polyadenylation site to +10 kb), and other (not in any of the four previous classes). The fractions expected from the respective sizes of the classes in the genome show that BMAL1 sites are mostly overrepresented in promoters and depleted inside genes. (D) Number of sites in close proximity (<10 kb) to annotated features, including non-coding RNAs (ncRNAs). These are split in micro RNAs (miRNAs) and others (long intergenic non-coding RNAs, small nucleolar RNAs, ribosomal RNAs, small nuclear RNAs, and miscellaneous RNAs). Middle column: fraction of sites from each category; last column: background fraction from the Ensembl annotation. The vast majority of sites (83%) are near genes expressed in liver (see Materials and Methods). (E) Strong BMAL1 binding correlates with high phylogenetic conservation. Sites are ranked according to the number of tags at their peak binding, and all Ensembl TSSs are shown as controls. In the window of ±50 bp around each site, the maximal value of the placental mammals PhastCons conservation score is used. PhastCons score ranges from 0 (no conservation) to 1 (perfect conservation). (F) Mean PhastCons conservation score for three classes of sites: 41 sites in RCGs (defined in Figure 1C), proximal sites (within 1 kb of an annotated Ensembl feature, 709 sites), and distal sites (1,340 sites). All categories are significantly more conserved than control regions (+500 bp downstream of each site). ***, *p*<1×10^−6^, Student's *t* test.

To study the location of BMAL1 binding sites relative to genes, we annotated each site with the nearest Ensembl transcript, including coding and non-coding genes. Positioning of BMAL1 sites with respect to the Ensembl annotation shows that 40% of the sites are within 1 kb, and 60% within 10 kb, of an annotated transcription start site (TSS) (Figure 2A) (random expectation is 15%, *p*<10^−16^, binomial test). Viewed on a finer scale, the 40% of sites within 1 kb of TSSs cluster slightly upstream of TSSs (50–100 bp upstream), while no similar correlation is observed for the 3′ ends of transcripts (Figure 2B). Compared to genomic frequencies, BMAL1 sites are strongly enriched in promoter regions (±2 kb around TSS) of coding genes and depleted inside genes (Figure 2C). To assess whether BMAL1 might also control non-coding genes, we considered all transcripts with a binding site within 10 kb and found that the majority of sites are close to coding genes (more than 50%), while few are found near RNA genes or microRNAs (Figure 2D; Text S2). Moreover, we found that BMAL1 binds in accessible and transcriptionally active chromatin regions, as 83% of the sites are located near genes that are expressed (defined as expressed above the median in RNA-Seq liver data [41]; Figure S3A; see Materials and Methods), which represents a highly significant fraction (*p*<10^−15^, rank test). Phylogenetic analysis showed that the conservation of BMAL1 sites increases with the strength of binding (Figure 2E), with the first 100 sites showing very high conservation (median PhastCons conservation scores near 1). Importantly, this is not simply a consequence of strongly bound sites tending to fall near TSSs (Figure S3B), as all Ensembl TSSs show lower conservation (Figure 2E). We further assessed conservation levels in both proximal sites (within 1 kb of an annotated Ensembl TSS) and distal sites, and found that both categories of sites were significantly more conserved than control regions (taken 500 bp downstream of each site), with distal sites showing on average less conservation than proximal sites (Figure 2F). On the same scale, sites close to RCGs showed strong conservation among mammalian species.

### BMAL1 Sites Are Associated with Carbohydrate and Lipid Metabolism, Transcriptional Regulation, and Cancer Pathways

Functional annotation analysis with DAVID [42],[43] identified enriched annotation clusters, the most prominent ones relating to carbohydrate and lipid metabolism, as well as transcriptional regulation in general (Table S2). This supports the finding that glucose metabolism is a major hepatic function directly controlled by BMAL1 [6],[7],[29],[38]. For example, glycolytic enzymes and transporters that were previously implicated in the circadian control of glucose homeostasis, e.g., *Pck1* and *Glut2* [7], as well as *G6Pase* [44], are identified as putative targets. As the mRNAs of these genes cycle with a phase that is expected for BMAL1/CLOCK targets, our data argue these key nodes are direct BMAL1 targets. Supporting this scenario, loss of function mutants have shown that BMAL1 and CLOCK are involved in glucose homeostasis [6],[45]. Similarly, lipid synthesis, notably sterol and triglyceride metabolism, is significantly enriched among BMAL1 targets, which substantiates the action of the core clock in these pathways. Interestingly, the most enriched functional cluster is transcriptional regulation: in total, 82 DNA transcription factors show BMAL1 binding, including 18 nuclear receptors, all expressed in liver (Table S3; [27]), 15 basic-leucine zipper proteins, 6 bHLH factors, and 10 zinc fingers (Table S3), indicating a hierarchic organization of circadian output programs. Notice, though, that only a minority of theses sites show binding strengths comparable to those of canonical clock genes. Unexpectedly, the *Bmal1* promoter itself shows a weak BMAL1 site, the significance of which is unclear at this point. More than 30% of these factors show rhythmic mRNA abundance on expression arrays (Table S3). To assess whether these factors are also circadianly active, we applied a bioinformatics analysis that combines known transcription factor consensus sites with mRNA measurements to infer active transcription factors [46],[47]. This method predicts a transcription factor as circadianly active when its putative targets, identified as those genes showing a conserved consensus binding site in their promoter, show phase coherent circadian expression (see Materials and Methods). Out of 22 factors with represented consensus sites, this analysis predicted circadian activity for those binding the DBP/HLF/TEF/E4BP4, REV-ERB/ROR, HIF1A, PPARα, and BACH1 consensus motifs (Figure S4), thus supporting a functional role for cyclic BMAL1 binding to the promoters of these regulators. Finally, enrichment of Kyoto Encyclopedia of Genes and Genomes (KEGG) pathways found cancer pathways as highly enriched in BMAL1 targets (DAVID, *p*<0.001; Table S4), notably in components of the cell cycle and in transforming growth factor beta (TGFβ) signaling (Figure S5). Specifically, we identify previously described [10],[11],[48]–[50] and novel links between the circadian clock and the cell cycle. For example, the G2-M-transition inhibitor *Wee1* is a putative target. Likewise, several cyclins of the G1-S transition (*Ccne1*, *Ccne2*, *Ccnd1,* and *Ccnd3*) and their partner, *cyclin-dependent kinase 4* (*Cdk4*), are also bound by BMAL1. Notably, several of these genes (e.g., *Ccnd2* and *Ccne2*) show circadian mRNA expression (Figure S5A). Other important pathways at the threshold of significance that have been previously linked to circadian function include the insulin [6],[45],[51],[52] and *Ppar*α [53]–[55] signaling pathways (Figure S5).

### BMAL1 Sites Are Enriched in E-Boxes and Tandem E1-E2 Elements

**Figure 3:**
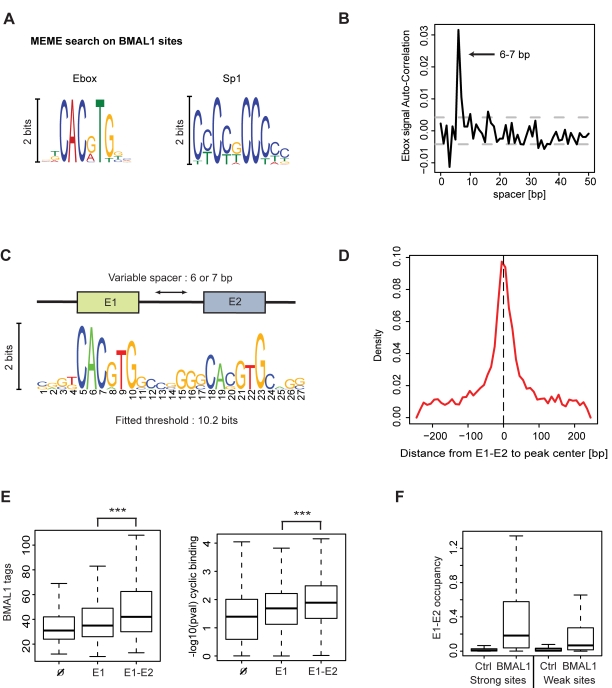
Genomic sequence preference of BMAL1 binding sites. (A) Overrepresented motifs found using MEME [56]. DNA sequences in the window of ±50 bp around each BMAL1 site were used for the sequence analysis. The enrichment of Sp1 sites reflects the proximity of BMAL1 sites to TSSs (Figure 2B). (B) Autocorrelation analysis shows that E-box motifs come in tandem, with a spacing of six or seven nucleotides. Grey dashed lines represent the 95% confidence interval. (C) HMM for the tandem E-box motif (E1-E2 element) converges to one canonical E-box site with threshold at 7.2 bits and a tandem E1-E2 element with threshold at 10.2 bits (Table S5). To train the model, each sequence was given a weight proportional to the number of BMAL1 tags at peak binding. (D) Distribution of distances from E1-E2 positions to peak centers. The E1-E2 elements are sharply located around the inferred binding location. (E) Sites with E1-E2 elements have significantly more tags (left) and show more robust rhythmic binding of BMAL1 (right) than sites without E-boxes (Ø) or with single E-boxes (E1). ***, *p*<5×10^−5^, Student's *t* test. (F) BMAL1 sites are strongly enriched in E1-E2 instances compared to control regions. Control regions were taken 500 bp downstream of each site.

Having discussed genomic positioning and functional annotations of BMAL1 sites, we aimed at refining current models for the DNA-binding specificity of BMAL1/CLOCK in vivo. To this end, we performed de novo motif searches and applied hidden Markov models (HMMs) to the genomic sequences surrounding the 2,049 binding sites. As expected, a MEME [56] analysis in short windows of ±50 bp around the predicted binding location (see Materials and Methods) clearly identified E-box signals as the strongest cis-element (Figure 3A). We also found an Sp1 motif, which is consistent with 40% of sites being located near TSSs [57] (Figure 2B). In the window considered, we did not identify other sequences that could indicate the involvement of further co-factors. On the other hand, a positional analysis of the E-box sequences indicates that these frequently occur in tandem with a spacer constraint of six or seven nucleotides (Figure 3B), reminiscent of the E1-E2 element [58],[59]. This configuration prompted us to train a nucleotide profile using a HMM that considers both single and variably spaced tandem elements (Figure S6B), similar to our previous model [58]. As the binding signal spans more than a decade (Figure 1D), sites bound by BMAL1 were weighted using the number of tags at peak binding for the training of the HMM. The sequence-specific profile converges toward two E-box elements, with inferred stringencies (cutoffs) that tolerate about one (E1) and three (E1-E2) mismatches (Figure 3C; Table S5). The genomic positions of the consensus sequences co-localize tightly with the predicted centers of the ChIP signals, i.e., they are mostly within ±25 bp (Figure 3D), which is largely because of the accuracy of the deconvolution method in localizing the binding sites. Overall, 13% of all BMAL1 sites had E1-E2 elements with spacers of 6 bp (7%) or 7 bp (6%), while in RCGs this fraction represented 29% of the sites, covering 53% of genes with at least one E1-E2 site. To investigate the influence of single and tandem E-boxes for BMAL1 binding, we divided the BMAL1 sites into three classes: sites with no E-box (Ø), sites with a single E-box (E1), and sites with E1-E2 elements (E1-E2). We found that E1-E2 sites have significantly more BMAL1 tags and more rhythmic binding profiles than E1 alone or empty sites (Figure 3E). Moreover, both strongly and weakly bound BMAL1 sites harbor significantly more E1-E2 elements than control regions taken 500 bp downstream of each site (Student's *t* test, *p*<2.2×10^−16^; Figure 3F). In summary, our sequence analysis showed that E1-E2 tandem repeats are overrepresented in BMAL1 sites and that the presence of such regulatory sites favors strong binding.

### E1-E2 Sites Are Bound Cooperatively by Dimers of BMAL1/CLOCK Heterodimers

**Figure 4:**
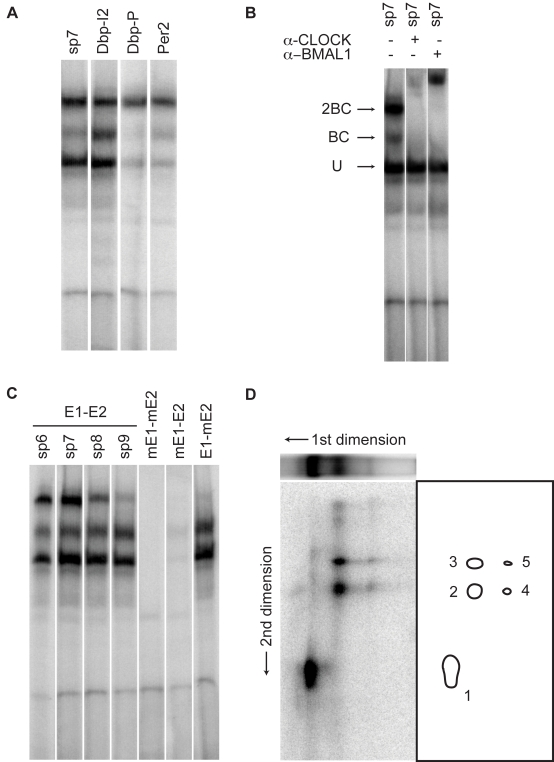
E1-E2 tandem sites are bound by dimers of BMAL1/CLOCK heterodimers. (A) EMSA gel analysis with nuclear extract from mouse livers harvested at the ZT2 time point. Extracts are incubated with oligonucleotides containing the naturally occurring E1-E2 sites in the *Dbp* promoter (Dbp-P), *Dbp* intron 2 (Dbp-I2), the *Per2* promoter (Per2), and an oligonucleotide with a canonical E-box (E1, CACGTG) and a non-canonical E-box (E2, AACGTG) spaced by seven nucleotides (sp7). Note: the shifts for the Dbp-I2 and sp7 probes are very similar. (B) Supershifts with anti-CLOCK and anti-BMAL1 antibodies identify two CLOCK- and BMAL1-containing complexes termed 2BC (heavier) and BC (intermediate weight), plus one unspecific complex (U, lowest weight). (C) Increased spacing and mutants. The upper 2BC band is reduced as the spacing between the E1 and E2 sites is increased from 6 bp (sp6) to 9 bp (sp9), the latter showing a pattern that resembles that obtained by mutating the E2-box but leaving E1 intact (E1-mE2). Mutating the canonical E-box (mE1-E2) strongly suppresses all complexes, while the doubly mutated probe (mE1-mE2) shows no binding. (D) Two-dimensional EMSA. The ZT2 extracts were incubated with sp7 oligonucleotides prepared with azido-dUTP nucleotides, and separated on a 1D EMSA gel (first dimension) (see Materials and Methods). The same three bands are found as in (A–C), and the weaker 2BC band compared to the regular probes (without the azido nucleotides) reflects reduced affinity following the azido substitutions in the E-box sites. In the second dimension, the five main spots indicate that the U complex contains one DNA-binding protein (spot 1), while the BC and 2BC complexes show identical DNA-binding constituents, namely CLOCK (96 kDa, spots 3 and 5) and BMAL1 (70 kDa, spots 2 and 4), as inferred by their approximate molecular mass.

The identified sequence elements prompted us to further characterize how BMAL1 complexes interact with DNA at these sites. We thus performed electromobility shift assays (EMSAs) with nuclear extracts from mouse livers. Using oligonucleotide probes from ChIP-Seq sites with E1-E2 sequences in the *Dbp* promoter, the *Dbp* intron 2, and the *Per2* promoter, we observed three main protein-DNA complexes, present in all probes (Figure 4A). Supershift assays with BMAL1 and CLOCK antibodies indicate that the two slowest migrating complexes, hereafter termed 2BC and BC, contain BMAL1 and CLOCK (Figure 4B). The supershift assay results also exclude the possibility of other DNA-binding complexes involving either one but not both. The third and fastest migrating complex most likely represents other E-box binding bHLH proteins expressed in liver, such as the abundant protein USF1, as discussed in [28]. Of the two BMAL1/CLOCK-containing complexes, 2BC shows stronger binding, which decreases when the spacing between the E1 and E2 sites increases (Figure 4C). In contrast, BC does not seem to be affected. This argues for a cooperative interaction between two BMAL1/CLOCK heterodimers at the E1-E2 sites that is reduced and eventually lost when the spacing increases. This is reflected in the pattern for the 9-bp spacer (sp9), which is comparable to that of a probe with an intact E1 site and a mutated E2 site (E1-mE2 probe). Finally, cross-linking protein-DNA complexes in combination with two-dimensional EMSA confirms that the BC and 2BC complexes have the same composition, i.e., they both contain CLOCK and BMAL1 but no other DNA-binding proteins (Figure 4D). Taken together, these data indicate a cooperative binding of two BMAL1/CLOCK heterodimers at E1-E2 elements.

### Naturally Spaced E1-E2 Sites Favor Strong Transcriptional Activation

**Figure 5:**
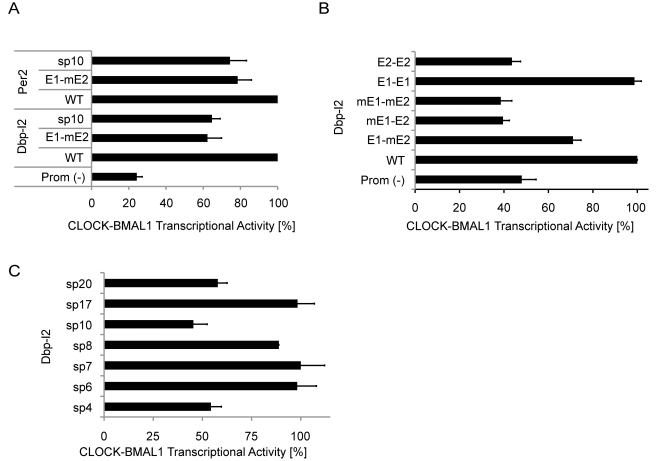
E1-E2 sites show increased transactivation compared to only E1 sites or E1-E2 sequences with longer spacers. (A) BMAL1-bound E1-E2 sites in the second intron of *Dbp* (Dbp-I2) and at the promoter of *Per2* show an increased BMAL1/CLOCK transactivation compared to either the E2-mutated (E1-mE2) version or the 10-bp spacer version (sp10) (Student's one-tailed *t* test, *p*<0.05, *n* = 3). Empty vector (Prom[−]) is shown as a negative control. Wild-type (WT) levels are set to 100%. Error bars represent the standard error of the mean. (B) Mutation in the Dbp-I2 sequence shows that an E1 is needed for robust BMAL1/CLOCK transactivation. The wild-type version was compared to E1 mutated (mE1-E2), both E1 and E2 mutated (mE1-mE2), E2 replaced by E1 (E1-E1), and E1 replaced by E2 (E2-E2). All mutated versions have reduced activity compared to wild-type (*p*<0.005, *n* = 4), with the exception of E1-E1, which shows a level of transactivation similar to that of wild-type. (C) Modifying the spacer length of the Dbp-I2 tandem E-boxes from 4 bp to 20 bp shows a spacer preference at 6–8 bp, but also at 17 bp, which corresponds to a full helical turn of the DNA. Indeed, sp4, sp10, and sp20 have a significantly reduced activity compared to sp7 (*p*<0.01, *n* = 4).

The data presented so far suggest that E1-E2 sites favor strong binding in vivo, which could result from cooperative binding of two BMAL1/CLOCK heterodimers at these elements. To substantiate the hypothesis that E1-E2 sites function as strong transcriptional enhancers, we performed transactivation assays by expressing BMAL1/CLOCK heterodimers in 293T cells and measured luciferase reporter constructs driven by wild-type E1-E2 sites from the *Dbp* intron 2 and *Per2* promoter sites, or by mutated sites with only one E1 site. In both cases, the constructs with only one E1 site (termed E1-mE2) show significantly reduced activity compared to the constructs with intact E1-E2 sites, namely about 50% for the *Dbp* and 70% for the *Per2* site (Figure 5A). Consistent with the EMSA results, reporter constructs with only the E2 site (mE1-E2 and E2-E2; Figure 5B) show transactivation levels comparable to background, underlining the importance of the E1 moiety. However, the E1-E1 construct had an activity similar to that of E1-E2, indicating that cooperativity can compensate for weaker binding affinity. When the spacing is increased from seven to ten nucleotides (sp10; Figure 5A), the activity is reduced to levels similar to those in E2 mutants (E1-mE2), suggesting that the interaction between the two BMAL1/CLOCK heterodimers is reduced when the phasing of the two binding sites is altered. According to this interpretation we observed that the transactivation increased again for spacers corresponding to one additional full helical turn of the DNA, i.e., spacers of 16–17 bp (Figure 5C). Notably, the intronic BMAL1 sites in *Rev-Erb*α and *Rev-Erb*β harbor a 16-bp E1-E2 element. These results thus argue that tandem E1-E2 sites play a role in determining the magnitude of BMAL1-dependent transactivation, which parallels our finding that such elements favor strong BMAL1 binding in the liver (Figure 3E).

### BMAL1 Targets Show Circadian mRNA Expression Profiles

**Figure 6:**
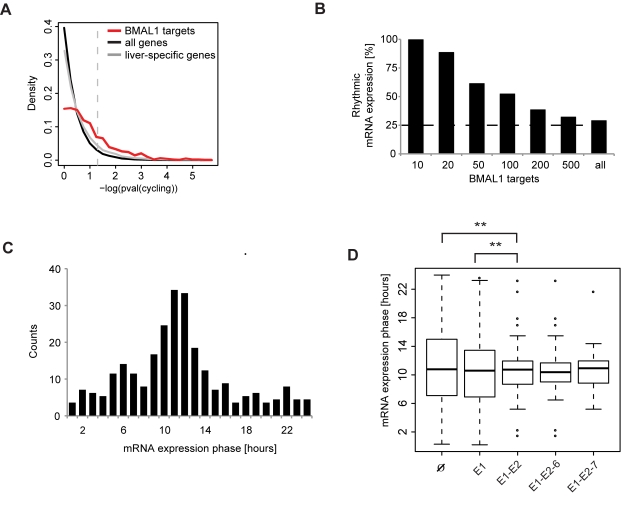
mRNA expression profiles of BMAL1 targets. (A) BMAL1 targets are highly enriched for robustly cycling mRNAs. Distribution of –log10 (*p*-value) are shown for all genes (black), liver-specific genes (see Materials and Methods) (grey), and BMAL1 targets (red). The vertical dashed line represents *p* = 0.05. Expression data are from mouse liver light-dark time course data [31]. BMAL1 sites were annotated with the closest protein-coding transcript in Ensembl within a window of 10 kb. (B) Strong BMAL1 binding is associated with circadian mRNA accumulation. The sites are ranked according to their strength, and the fraction of robustly circadian mRNA patterns (Fisher test, *p*<0.05; see Materials and Methods) among the top *x* sites is shown. This fraction decreases from 100% among the top 10 sites to 29% for all sites, and this proportion is practically unchanged (31%) if we restrict our analysis to only rhythmic BMAL1 sites. (C) The distribution of the phase of cytosolic mRNA expression of targets of BMAL1 peaks at ZT10-ZT12. (D) BMAL1 targets with an E1-E2 element show narrower mRNA phase distribution than targets with no or single E-boxes (Rao homogeneity test for circular data [60], *p*[equality of dispersions]<0.01). E1-E2-6 and E1-E2-7 represent, respectively, the subgroup of E1-E2 sites with a spacer of 6 bp and 7 bp.

Our positional analysis of BMAL1 sites showed that more than 60% are located less than 10 kb from a TSS, which was emphasized by the strong enrichment of sites in promoter regions (Figure 2B). To assess whether BMAL1 binding near coding genes, i.e., located less than 10 kb from a TSS, is predictive of a circadian mRNA expression pattern and to determine a possible functional role for the E1-E2 element, we compared the putative targets with mRNA expression profiles in liver sampled around the clock [31]. The set of BMAL1 targets was highly enriched (*p*<2×10^−16^, two-sample Wilcoxon test) in circadian mRNA profiles (Figure 6A), also when we restricted our analysis to liver-specific genes (see Materials and Methods), excluding the possibility that this would merely reflect the numerous circadianly expressed transcripts in liver. Stratifying the analysis according to binding strength, we found that strong binding is highly predictive of rhythmic mRNA expression. Namely, for all BMAL1 sites with a TSS within 10 kb, 100% of targets robustly cycled among the top 10, 85% among the top 20, over 50% among the top 100, and 29% in total (Figure 6B). Consistent with the maximal binding of BMAL1 around ZT6 (Figure 1A and 1B), the expression phase of the rhythmic targets peaked around ZT10 (Figure 6C). Interestingly, the distribution of expression phases in targets with or without E1-E2 elements differed significantly: although targets harboring E1-E2 elements showed a similar mean phase compared to targets without or with single E-boxes, these genes showed a tighter mRNA phase dispersion (Rao homogeneity test for circular data [60], *p*<0.01), suggesting a role for the E1-E2 element in controlling the precision of the circadian expression phase (Figure 6D). However, we did not observe a significant difference in the mean nor in the dispersion between the 6-bp and 7-bp spacer variants of E1-E2. Therefore, these results suggest that a fair fraction of the BMAL1 sites induce rhythmic transcription, and that E1-E2 elements play a role in the precise temporal expression of BMAL1 targets.

### Transcriptional versus Post-Transcriptional Control of mRNA Expression

**Figure 7:**
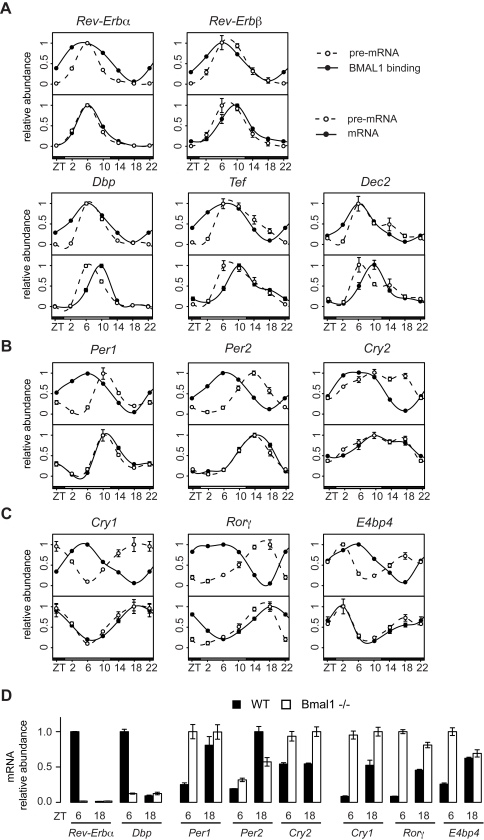
Phase relationships between BMAL1 binding, pre-mRNA, and mRNA accumulation. (A–C) BMAL1 binding profiles (filled symbols, upper panels) in comparison to qPCR measurement on pre-mRNA (open symbols) and mRNA (filled symbols, lower panels). The clock genes are separated into three groups based on the difference of phase of pre-mRNA expression and BMAL1 binding. The data represent the mean ± standard deviation of three experiments. The maximal value was normalized to 1. ZT22 is plotted twice to facilitate visualization. (A) Early targets. *Rev-Erb*α, *Rev-Erb*β, *Dbp, Tef,* and *Dec2* pre-mRNA accumulation coincides with the BMAL1 binding profile. (B) Intermediate targets. *Per1*, *Per2,* and *Cry2* pre-mRNA accumulation is delayed by a few hours relative to the BMAL1 binding profile. (C) Late targets. For *Cry1*, *Ror*γ, and *E4bp4*, BMAL1 binding does not predict pre-mRNA accumulation. (D) mRNA expression levels in wild-type (WT) and *Bmal1* ^−/−^ mice. Expression levels were measured at ZT6 and ZT18. The clock genes are separated into three groups as in (A–C). Early targets are likely to be controlled directly and only by BMAL1 since their mRNA levels are low both at ZT6 and ZT18 in *Bmal1* ^−/−^ mice. Intermediate and late targets have either intermediate or elevated mRNA levels in *Bmal1* ^−/−^ mice, suggesting more complex transcriptional regulation. The data were analyzed as in (A–C).

To further study the temporal relationship between rhythmic BMAL1 binding, transcription, and mRNA accumulation, we quantified temporal profiles of pre-mRNA and mRNA for canonical clock genes showing strongly rhythmic BMAL1 sites. Comparing the profiles of BMAL1 binding and pre-mRNA accumulation identified several outcomes: (i) for early genes, *Rev-Erb*α, *Rev-Erb*β, *Dbp*, *Tef,* and *Dec2*, the pre-mRNA closely follows binding without significant delay, suggesting that transcription largely depends on BMAL1/CLOCK (Figure 7A); (ii) genes such as *Per1*, *Per2,* and *Cry2* show pre-mRNA accumulation levels that are delayed by less than 4 h compared to BMAL1 binding, suggesting that other regulators contribute to transcription (Figure 7B); (iii) finally, *Cry1*, *Ror*γ, and *E4bp4* show pre-mRNA accumulation profiles that are delayed by about 12 h, indicating that other regulators are dominant in determining the phase of transcription (Figure 7C). The mRNA profiles followed pre-mRNA accumulation with short delays of maximally 4 h (see *Dbp*, *Tef,* and *Dec2*). We expected that longer lived mRNA transcripts would show delayed phase and reduced amplitude compared to pre-mRNA profiles, which was supported by the *Gys2*, *March8,* and *Qdpr* genes (Figure S7A); proxies for mRNA half-lives from mouse embryonic stem cells [61] and fibroblasts [62] showed consistency in these cell types (Figure S7B). To test the prediction that transcription in early targets depends largely on BMAL1, while additional regulators contribute to the other cases, we compared mRNA accumulation for several genes in wild-type and *Bmal1^−^* ^/*−*^ animals at both peak (ZT6) and trough (ZT18) BMAL1 activity time points (Figure 7D). We found that the expression of the early genes, *Rev-Erb*α and *Dbp*, was strongly suppressed in *Bmal1* ^−/−^ mice. Moreover, genes of the intermediate or late types showed similar (e.g., *Per2*) or higher (e.g., *Per1*, *Cry1*, *Cry2*, *Ror*γ, and *E4bp4*) levels of expression in *Bmal1* ^−/−^ compared to wild-type, indicating that for these categories, *Bmal1* can act as a repressor, either directly [63] or indirectly [15]. Our *Bmal1* ^−/−^ mRNA data are consistent with measurements obtained at different time points in light-dark time courses [64], and for dark-dark time courses [15]. For *Tef,* the former data indicate a regulation in between early and intermediate types. Taken together, these results show that the phase of BMAL1 binding explains temporal accumulation of the early circadian transcripts. In addition, genes with delayed pre-mRNA profiles indicate that other circadian regulators contribute to transcription. Therefore, additional data for circadian activators and repressors will be key to further dissecting the transcriptional logic by which the binding amplitudes and phases of such regulators are integrated at circadian promoters.

## Discussion

### Widespread Rhythmic and Phase-Specific Binding of BMAL1 in Mouse Liver

Circadian gene expression relies on rhythmic transcription mediated by transcription factors, among which is the master regulator BMAL1/CLOCK in mammals. In this study, we identify more than 2,000 sites, of which 60% are rhythmically bound by BMAL1 in mouse liver under physiological light-dark conditions (Figure 1B). As liver tissue is mainly entrained through circadian signals from the suprachiasmatic nucleus or from feeding cues, we expect little differences with dark-dark conditions. Nevertheless, future studies in dark-dark conditions will allow estimating the changes in BMAL1 binding that are strictly dependent on the core clock. Our results substantiate at the genome-wide level the model [24],[65],[66] that rhythmic protein-DNA interactions in mammals underlie phase-specific circadian gene expression, which is reminiscent of widespread circadian binding found for dCLK in *Drosophila* [67], or the circadian WHITE COLLAR COMPLEX (WCC) in *Neurospora* [68]. Importantly, we found that peak BMAL1 binding is fairly narrowly centered around ZT6, indicating that it does not contribute much to flexibility in specifying phase at this regulatory level. As BMAL1 can form functional bHLH heterodimers with CLOCK and NPAS2 [12],[13], our data do not distinguish between targets specific for either partner. In liver, NPAS2 protein is weakly expressed [69]; however, our EMSA analysis (Figure 4B) with liver extracts did not indicate that putative BMAL1/NPAS2 complexes bind E-boxes or tandem E1-E2 elements. Similarly the BMAL1 paralog BMAL2, which is very weakly expressed in liver at the mRNA level [41],[70], can form functional BMAL2/CLOCK dimers [71]–[73] but those are not recognized by our antibody, which is highly specific to BMAL1 (Figure S8). Interestingly, we find that strongly bound BMAL1 sites exhibit high phylogenetic conservation among placental mammals, which is even more pronounced in RCGs. As recent studies showed that CEBPA and HNF4A binding in the liver could be highly species-specific [74], it would be interesting to compare our results with BMAL1 ChIP data from livers in other mammalian species.

### Circadian Clock Genes Are the Strongest BMAL1 Target Genes

**Figure 8:**
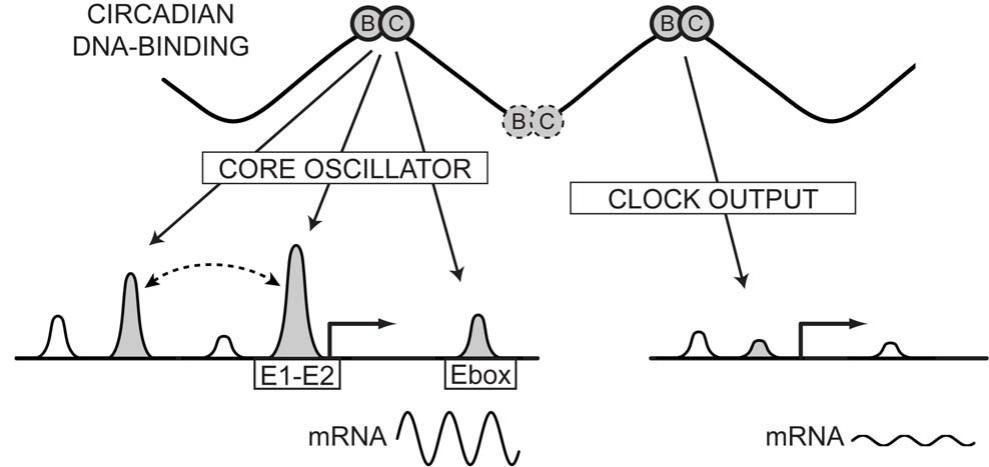
Cooperative interactions drive strong circadian amplitudes: a hypothetical model. BMAL1 rhythmically binds thousands of sites in liver, with peak binding around ZT6. Among the targets, core oscillator genes stand out as the strongest and often exhibit multiple BMAL1 binding sites. E1-E2 elements favor strong binding and precise phase-specific gene expression. The many weaker sites are distributed among clock output programs in liver, notably carbohydrate and lipid metabolism. A hypothesis for the differential binding and circadian amplitude of mRNA outputs between core oscillator genes and clock outputs is that strong sites use cooperative interactions with other regulators, or between multiple BMAL1 sites.

Surprisingly, the distribution of binding strengths showed relatively few (<50) sites with binding strengths comparable in magnitude to those of core circadian genes. This indicates that BMAL1 plays a major transcriptional role in the core oscillator, while the many weaker sites suggest that it controls diverse output programs in a more distributed fashion. Among the strongest targets, known circadian genes are indeed largely overrepresented, and we found that many bona fide regulatory elements for BMAL1/CLOCK, e.g., those in *Dbp* [24],[62], *Per2* [71], and *Per1/2/3* [59],[72], were strongly bound by BMAL1. Several of those elements, e.g., in *Dbp* or *Per2*, contain previously identified E1-E2 elements [58]. However, this selectivity cannot be explained by sequence-specific binding alone. Although strongly bound sites are enriched in E1-E2 consensus sites, we also find sites with such elements that are bound more weakly (Figure 3E). As the measured ChIP signal is determined by a combination of sequence-specific binding, cooperative interaction with co-regulators, and chromatin accessibility, it is difficult to determine what distinguishes strong from weaker sites. We have just argued that sequence specificity is only partially informative, and differences in accessibility are also unlikely, as we showed that 83% of the sites fall near expressed genes. Thus, it may be that yet uncharacterized cooperative interactions with co-regulators, or cooperative interactions between multiple BMAL1 sites, are primarily responsible for the strong binding at core circadian genes (Figure 8). One candidate co-regulator could be the SP1 protein, which was suggested to bind DNA circadianly [37], and also found as an enriched cis-element (Figure 3A). Supporting the scenario of multiple interacting BMAL1 sites, we found that circadian genes often contain multiple BMAL1 binding sites (Figure S2), which could be involved in long-range DNA interactions, as proposed for the estrogen receptor [75].

### Sequence Specificity of BMAL1/CLOCK Complexes

Previous bioinformatics analyses, including our own, identified evolutionarily conserved E-boxes and E1-E2 sites as putative BMAL1/CLOCK consensus sites in vertebrates [58],[59],[76], both of which were shown to drive rhythmic transcription in luciferase reporter assays [59],[76]. Here we established in vivo that both simple and tandem E-boxes are characteristic of BMAL1 target genes. While the sites comprising E1-E2 elements are overall in the minority, these sites contain a number of distinguishing features: (i) more than half of the RCGs have such sites bound in vivo; (ii) E1-E2 sites are associated with strong and rhythmic binding (Figure 3E); (iii) finally, the comparison with microarray data indicates that E1-E2 sites show comparably tighter mRNA expression phases (Figure 6D). Our in vitro experiments show that BMAL1/CLOCK binding to E1-E2 elements involves a cooperative and spacing-dependent interaction between the tandem sites, consistent with the constraint in the spacer length that was identified computationally [58],[59]. Together, our data argue that single E-boxes in the genomic context are sufficient to recruit BMAL1/CLOCK heterodimers rhythmically, while E1-E2 elements may play a role in the core clock to ensure precise ticking of the circadian clock.

### Does Circadian BMAL1 Binding Predict the Timing of mRNA Accumulation?

A central question was to study the relationship between circadian DNA binding and mRNA expression. Although the nature of ChIP experiments does not imply that circadian oscillations in DNA binding necessarily lead to a circadian modulation of the transcription rates, the body of experiments and analyses shown here indicate that a large fraction of the BMAL1 sites lead to circadian modulation in transcription. For instance, a significant fraction of BMAL1 targets show robustly circadian mRNA expression, with a peak phase that is delayed by a few hours compared to peak BMAL1 binding. Indeed, the analysis of binding profiles shows that BMAL1 binding is mainly restricted to ZT4–ZT8, while the phases of mRNA expression are centered at ZT10–ZT12, with a distribution that is broader than that of binding (Figure 6C). Analysis of pre-mRNA and mRNA levels of core clock genes in wild-type and mutant *Bmal1* ^−/−^ animals indicated that transcription of genes with early phases (in phase with BMAL1 binding) depended predominantly on BMAL1, while that of delayed genes involved further regulators. Other regulators that have been implicated in the tuning of circadian expression phase include the DEC [77] and CRY [66] repressors. The finding that delayed genes tended to be upregulated in the knockout condition suggests that BMAL1 could act as a repressor either via direct [63] or indirect mechanisms [15], as has been previously proposed. While the genetic data [15] indicate that the delays reflect a primary regulation by the *Rev-Erb/Ror* repressor/activator pair, we showed that these genes nevertheless do have rhythmically bound BMAL1 binding sites. Moreover, the timing of mRNA expression can also be influenced by post-transcriptional mechanisms that regulate the stability of the transcripts, such as those mediated by microRNA. In fact, transcript stability affects not only the phase but also the amplitude of the mRNA accumulation. If the amplitude of the pre-mRNA is weak already, a long mRNA half-life can cause the mRNA accumulation to be practically constitutive, as exemplified by *March8* mRNA levels (Figure S7A). For this reason, the fraction of cyclic mRNA transcripts among BMAL1 targets probably underestimates the fraction of functional sites, i.e., those that drive rhythmic transcription.

### A Hierarchy of BMAL1-Controlled Metabolic Functions and Gating of the Cell Cycle

The large number of transcriptional regulators among putative BMAL1 targets emphasizes the pervasiveness of the circadian oscillator in liver function and shows the hierarchical control of circadian output function. Accordingly, circadian transcriptional regulators controlled by BMAL1/CLOCK can transmit their phase information to downstream targets, a model that is supported by regression analyses that predict circadian activity for several of those targets (Figure S4). These findings substantiate regulatory links that were proposed in previous computational studies aimed at reconstructing the circadian transcription regulatory network [78]–[80]. In our ontology analysis, nuclear receptors appeared as the most overrepresented annotation cluster, which may reflect their role in serving as a relay between the circadian clock and metabolic processes [27],[81], as well as in orchestrating tissue-specific circadian gene expression [30]. BMAL1 also appears to directly control specific pathways such as glucose metabolism (*Gys2*, *Glut2,* and *Pck1*) and triglyceride metabolism (*Insig1*/*2* and *Pnpla2*). This dual, direct and indirect, regulation of circadian output function is emphasized by the presence of feed-forward loops (FFLs) [82] among targets, and might be implicated in the control of circadian expression phase. For instance, BMAL1 binds P450 oxydoreductase (*Por*), which was previously identified as a DBP/HLF/TEF target [5] with robust cyclic mRNA expression [7]. Similarly, BMAL1 binds both *Hif1*α and its known target *Vegfa* (Figure S5A). Interestingly, HIF1α, which we also predicted to be circadianly active (Figure S4), has been previously linked to the circadian clock as a CLOCK interacting protein [83] and in large-scale small interfering RNA perturbation experiments [47]. A number of studies have suggested that transcriptional regulation of cell cycle components by the circadian clock would lead to temporal gating of cell division [10],[11],[48]–[50]. Our data provide a number of additional links between these processes, in particular for regulators of the G1-S transition. Therefore, the circadian clock appears to not only interact with the cell cycle at G2-M [10] but might also influence entry into S phase.

### Conclusions

In conclusion, our circadian time course ChIP analysis showed that BMAL1 binds over 2,000 sites in the mouse genome. In addition, we found highly phase-specific binding patterns, peaking at ZT6. The distribution of binding strength rapidly decays, i.e., we find at most a few dozen sites with magnitudes in the range of those found at core oscillator genes or PAR-bZip transcription factors. This strengthens the idea of BMAL1's primary function as master regulator of the circadian clock, with weaker contributions to a variety of output programs. At the genomic sequence level, strong sites also more frequently harbor highly conserved tandem E1-E2 sites, and the latter are bound cooperatively by dimers of BMAL1/CLOCK heterodimers. Genes with such elements also showed more tightly distributed phases in their mRNA expression. However, while some genes are principally regulated by BMAL1/CLOCK, other targets exhibit more complex temporal patterns in their precursor and mature RNA, hinting at contributions from further regulators. The large number of transcription factors among BMAL1 targets is reminiscent of the hierarchic organization of circadian output pathways in mouse liver. This network structure may provide flexibility in the control of tissue-specific output programs by peripheral oscillators.

## Materials and Methods

### Animals

Animals were housed under a 12-h light/12-h dark regimen with food and water available ad libitum. ZT0 is defined as the time when the lights are turned on. Animals were housed for 3 wk under the indicated photoperiods. The age of the animals was between 3 and 4 mo. All animal care and handling was performed according to the State of Geneva's law for animal protection.

### Chromatin Immunoprecipitation

For each time point, livers from two mice were pooled to prepare chromatin as in [24]. For the BMAL1 ChIP, a polyclonal anti-rabbit antibody to a C-terminal peptide was raised and purified using standard techniques. The specificity of the antibody for BMAL1 was ascertained using SDS-PAGE with nuclear extract from wild-type and *Bmal1* ^−/−^ animals (Figure S8); extracts were provided by Frédéric Gachon (University of Lausanne). Sepharose-protein A beads (GE Healthcare) were prepared according to manufacturer indications and resuspended in RIPA buffer (50 mM Tris-HCl [pH 8], 150 mM NaCl, 2 mM EDTA [pH 8], 1% Triton X-100, 0.5% sodium deoxycholate, 0.1% SDS) supplemented with Roche Complete Protease Inhibitor Cocktail. Chromatin (250 µl) was pre-cleared by incubating with 60 µl of bead suspension for 1.5 h at 4°C on the rotating wheel. Pre-cleared chromatin was then incubated with 4 µl of BMAL1 antibody for 5 h at 4°C on the rotating wheel. Bead suspension (35 µl) was added to each reaction, and incubation was continued for 3 h at 4°C on the rotating wheel. Beads were then washed three times with wash buffer (0.1% SDS, 1% Triton X-100, 2 mM EDTA [pH 8], 150 mM NaCl, 20 mM Tris-HCl [pH 8]) and once with final wash buffer (0.1% SDS, 1% Triton X-100, 2 mM EDTA [pH 8], 500 mM NaCl, 20 mM Tris-HCl [pH 8]). Co-immunoprecipitated DNA fragments were eluted from the beads in 120 µl of 1% SDS and 100 mM NaHCO_3_ for 15 min at 30°C and then treated with 1 µl of RNase A for 1 h at 37°C. Co-immunoprecipitated DNA fragments were incubated overnight at 65°C with Proteinase K and then purified using Qiaquick PCR Purification Kit (Qiagen). For real-time PCR quantification, the equivalent of 5 µl of chromatin of each reaction was used in a 20-µl reaction using the primers and TaqMan probes listed in Table S6, using an ABI 7900HT PCR machine (Applied Biosystems). For Illumina sequencing, two sets of libraries were prepared with independent BMAL1 ChIP time courses (library A: one time course; library B: pool of three time courses), and a total of 16 lanes were sequenced on an Illumina Genome Analyzer 2 machine. To prepare the input library, samples from the six time points were pooled at equal amounts, and one lane was sequenced.

### ChIP-Seq Data Analysis

At each time point, sequenced DNA reads from both libraries were pooled and mapped to the mouse genome (*Mus musculus* National Center for Biotechnology Information m37 genome assembly [mm9; July 2007]) using Bowtie [84] with three mismatches and only one hit allowed on the genome. If several reads coming from the same library mapped at the same genomic position and on the same strand (redundant tags), we considered this as a PCR duplicate and only one read was kept for the rest of the analysis. The numbers of reads per time point are shown in Table S1. To normalize for differences in sequencing depth among the time points, the number of tags per position in each BMAL1 ChIP-Seq library was rescaled by the total number of mapped tags in this library, and then for each time point, the numbers of tags in each library were summed up. The number of tags in a binding site is expressed as the number of non-redundant tags per 10^7^ aligned tags, with the best sites in the range of 200. The list of all sites with their annotations is given in Text S2.

### Peak Identification

At each time point separately, BMAL1-bound regions were detected by MACS [36] with the following parameters: shift = 75, bandwidth = 150, genome size = 2.4 Gb, and the input chromatin sample as control data; overlapping binding regions were merged. In each region, a refined estimate of the binding location was obtained using a deconvolution algorithm that models the expected distribution of tags on the positive and negative strands (see Text S1). This was done on a single track in which all tags from all time points were merged. Local maxima in the deconvolved signal were used to call binding site positions for the rest of the analysis. The deconvolution methods also allowed us to efficiently reject spurious sites, leaving us with a total of 2,049 trustable binding sites. For each binding site, the signal in windows of ±250 bp were quantified for each time point and subjected to rythmicity analysis.

### Binding Site Annotation

Each binding site was annotated with the Ensembl transcript having the closest TSS using the R package biomaRt. The Ensembl transcript ID was then used to retrieve further annotations such as Mouse Genome Informatics symbol, Entrez Gene ID, and Affymetrix Mouse 430 probe ID. Mouse liver RNA-Seq data from [41] were used to define the liver transcriptome (threshold was set at 1.35 reads per kilobase per million mapped reads, corresponding to the 50^th^ percentile; see Figure S3A).

### Sequence Analysis

DNA sequences and placental mammals PhastCons conservation scores [85] in windows of ±50 bp around the center of each binding site were retrieved from Ensembl and the UCSC Genome Browser database, respectively. To analyze correlations in the positions of E-boxes, the sequences for all BMAL1 binding sites were scanned with a weight matrix (Figure S6A), and the resulting likelihood scores were converted to occupancies using a sigmoid transformation with threshold corresponding to one mismatch. The correlation signal was then computed on the occupancies. The HMM was trained using the sequences under all BMAL1 binding sites, weighted proportionally to the number of BMAL1 tags at peak binding, using the model architecture shown in Figure S6B. To compute the position of E1-E2 instances with respect to binding sites, we extracted weight matrices from the trained HMM with spacing ranging from 6 to 7 bp and scanned windows of ±250 bp around each binding site.

### Fourier Analysis and Microarray Data

Time series expression data were from [31] using the plus-doxycyclin condition, which mimics wild-type light-dark conditions. Liver-expressed genes for these data were defined as having mean log2 (expression) over the 12 time points greater than 3.5 (Figure S3A). The 24-h Fourier component (F24) and phase were computed using established methods [86], and the *p*-value associated with 24-h rhythmic expression (also for cyclic binding) was computed using a Fisher test for one specific period [87] for a time series at 4-h intervals of even length *N*:
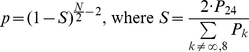
(1)


### Inference of Transcription Factor Activities

Times series data as above [31] were combined with position-specific weight matrices (PSWMs) from the SwissRegulon database [47] to predict transcription factor activities using a regression model similar to that in [47]. Briefly, we fitted the following multi-linear model:
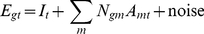
(2)where *E_gt_* is the mean-centered expression level of the gene *g* at time *t*, *N_gm_* is the number of predicted conserved sites for motif *m*, and *A_mt_* is the activity of the motif *m* at time *t*. *I_t_* denotes an intercept. To compute the *N_gm_* matrix, windows of ±2,500 bp around each Ensembl transcript annotated with an Affymetrix Mouse 430 probe ID were scored with the corresponding PSWM, and the likelihoods along the sequence were summed up [46] and weighted by a factor *C* ^0.05^, where *C* stands for the product of the PhastCons conservation scores in that sequence. Given expression data *E_gt_* and the occupancies *N_gm_*, the unknown activities *A_mt_* are then inferred using standard least squares regression.

### RNA Isolation and Analysis

To quantify pre-mRNA and mRNA levels with real-time RT-PCR, whole cell RNA was isolated according to [88]. For each time point, the extracted RNA from four livers was pooled (in each case two of the four livers were from the animals used for the chromatin preparation). For the *Bmal1* ^−/−^ samples at ZT6 and ZT18 (provided by Frédéric Gachon), total RNA from two livers was pooled. Pooled RNA (0.5 µg) was reverse-transcribed using random hexamers and Superscript reverse transcriptase (Invitrogen). The cDNA equivalent to 20 ng of total RNA was PCR-amplified in an ABI 7900HT PCR machine using the primer and TaqMan probes listed in Table S7. The relative levels of each RNA were calculated on the basis of 2^−CT^ and normalized to the corresponding levels of *Gapdh* RNA. Each mRNA time course was normalized by its mean value, and the data shown represent the mean±standard deviation of three independent time courses.

### Electromobility Shift Assays

EMSA and preparation of nuclear extracts were performed as in [37] with the following modifications. EMSA probes were prepared by dissolving forward and reverse oligonucleotides (listed in Table S8) in 100 mM NaCl, annealing them by warming them to 95°C and letting them cool down to 25°C over the course of several hours. Annealing oligonucleotides (30 µl, 25 ng/µl) were incubated with 4 µl of Klenow fill-in buffer, 2 µl of 5 mM dATP/dGTP/dTTP, 2 µl of 3,000 Ci/mmol 32-dCTP, and 2 µl of 5 U/µl Klenow fragment for 15 min at room temperature. Radiolabeled probes were then purified using Qiaquick Nucleotide Removal Kit (Qiagen) and resuspended in 15 µl of H_2_O. For supershift experiments, 1 µl of purified antibody was added immediately before the addition of the radioactive probe. The antibodies used were anti-BMAL1 and anti-CLOCK from [28]. Two-dimensional EMSA was performed as in [28] with the following modification: the protein-DNA complexes were separated on a 4% acrylamide gel by electrophoresis (first dimension).

### Transactivation Assays

293T cells were cultured in Dulbecco's Modified Eagle Medium supplemented with 10% fetal bovine serum (Invitrogen) and 1.5% streptomycin/penicillin antibiotics (Cellgro) under 5% CO_2_ at 37°C. Twenty-four hours after seeding at 1.5×10^5^ cells/ml, cells were transfected using LipofectAMIN 2000 (Invitrogen). At 28 h after transfection, cells were harvested, and the luciferase activity was determined by using Dual Luciferase Reporter Assay (Promega) on a luminometer (EnVision 2104 MultiLabel Reader, PerkinElmer). Transactivation assays were performed using 1,200 ng of total DNA per well (300 ng of pDEST26-BMAL1, 300 ng of pDEST26-CLOCK, 50 ng of different pGL3-Promoter constructs [firefly luciferase], phRL-SV40 [renilla luciferase]) and a total of 1,200 ng of pDEST26-LACZ plasmids. Different E-box motifs were inserted upstream of the SV40 promoter of pGL3-Promoter vector (Promega) by using annealed primers (Table S9) and ligated into KpnI-XhoI sites.

### Data Availability

Illumina sequencing data for the BMAL1 ChIP are available at Gene Expression Omnibus (http://www.ncbi.nlm.nih.gov/geo/), accession number GSE26602. Processed BigWig files that can be visualized on the UCSC Genome Browser as a custom track to generate graphs such as Figure 1D and 1E are available at http://circaclock.epfl.ch. The fully annotated (including binding strength) 2,049 sites are provided in Text S2.

## Supporting Information

Figure S1 **BMAL1 ChIP-qPCR at control loci.** Two positive control loci, the *Per1* promoter (A) and the *Dbp* site in intron 2 (B), show circadian BMAL1 binding. Fold enrichments relative to *glyceraldehyde 3-phosphate dehydrogenase (Gapdh)* are greater than 100-fold at ZT6 and about 10-fold at ZT18.
(0.33 MB PDF)

Figure S2 **ChIP-Seq time series at circadian reference genes.** Data viewed in the UCSC Genome Browser showing two BMAL1 sites in *Cry1* (A), two in *Cry2* (B), two in *Dec1* (C), four in *Dec2* (D), three in *E4bp4* (E), three in *Hlf* (F), five in *Per1* (G), two in *Per2* (H), one in *Rev-Erb*β (I), one in *Ror*γ (J), and four in *Tef* (K) loci. RefSeq annotation and PhastCons placental mammal conservation score are displayed.
(2.60 MB PDF)

Figure S3 **Liver RNA-Seq data define the liver-specific transcriptome, and binding strength depends on distance to genes.** (A) Defining the liver-specific transcriptome from RNA-Seq data. Number of reads per kilobase per million mapped reads (RPKM) from RNA-Seq data [41] correlates with microarray data (normalized with RMA) averaged over time points from [31]. Liver-expressed genes were defined as genes with more than 1.35 reads per kilobase per million mapped reads (red line, 50% percentile). (B) Stronger BMAL1 sites are located closer to TSSs than weaker sites. The sites are binned according to rank, as in Figure 2E.
(0.94 MB PDF)

Figure S4 **Inferring transcription factor activities from linear regression models.** Inferred activity profiles (*A_mt_*) for BMAL1 targets (see Materials and Methods). Microarray data from [31] were used together with the PSWMs of the corresponding transcription factors (SwissRegulon database) to infer motif activities. Logo of the PSWM is shown above each profile, and phase of peak activity is indicated. Grey shades represent the standard errors of the linear regression at each time point. Only profiles with cyclic activity profiles are shown (Fisher test, *p<*0.05; see Materials and Methods). RRE stands for ROR response element, D-box is the DBP consensus element, PPARα is the *Ppar*α binding site, HIF1A is a bHLH regulator, and BACH1 is a CNC-bZip leucine zipper protein.
(0.50 MB PDF)

Figure S5 **BMAL1 targets in KEGG pathways.** (A) BMAL1 targets in the “Pathways in Cancer” KEGG pathway. Targets are colored according to phase of their mRNA expression in the data from [31]. The color legend is given in (C). All targets are shown as red boxes, but only those with well-defined phases (F24>0.2) are colored. (B) BMAL1 targets in the insulin signaling pathway. (C) BMAL1 targets in the *Pparα* signaling pathway. These graphs were generated using KEGG Mapper (http://www.genome.jp/kegg/tool/color_pathway.html).
(1.31 MB PDF)

Figure S6 **Weight matrix and structure of the HMM used for sequence analysis.** (A) Logo of the E-box PSWM used for autocorrelation analysis. At each position of the PSWM, the most probable letter has *p* = 0.96875, while the others have *p* = 0.03125. (B) Structure of the HMM. E1 and E2 model, respectively, the collection of hidden states of the first and second E-box. M states allow for filtering of spurious signal, namely GTGT repeats. B1 and SP represent, respectively, background and spacer states. For simplicity, the reverse complement of the HMM is not shown here.
(0.35 MB PDF)

Figure S7 **Pre-mRNA and mRNA measurements of longer lived transcripts.** (A) mRNA transcript stability may explain lag and relative amplitude between pre-mRNA and mRNA accumulation in the *Gys2*, *March8,* and *Qdpr* transcripts. Experiments were performed as described in Figure 7A–7C. Approximate half-lives for *March8* and *Qdpr* are 5.4 h and >10 h, while that for *Gys2* is not available (see [B]). (B) mRNA half-lives from mouse embryonic stem cells [61] and mouse fibroblasts [62] for the genes in Figure 7A–7C and (A). When several measurements from the same cell line were available, we took the mean.
(0.51 MB PDF)

Figure S8 **The anti-BMAL1 antibody recognizes specifically BMAL1.** Ponceau staining (A) and Western blot (B and C) of nuclear extracts (15 ug) from wild-type and *Bmal1* ^−/−^ mouse liver at ZT6. The nuclear extracts were electrophoresed on a 12% SDS-PAGE gel, transferred onto a nitrocellulose membrane, and detected using anti-POLII Cter (ab817-100, Abcam) (B) and anti-BMAL1 antibodies (C). The sequence of the peptide used for the immunization is located at the C-terminal of the mouse BMAL1 protein: LEADAGLGGPVDFSDLPWPL.
(3.48 MB PDF)

Table S1 **Sequencing data: number of sequenced and non-redundant tags at each time point.**
(0.05 MB PDF)

Table S2 **Functional annotation clustering of putative BMAL1 targets using DAVID tools.** These annotations link the sites to the closest gene irrespective of the distance. In total, 1,551 out of 2,049 sites have a functional annotation. For details regarding the positions and binding strength of these sites, see Text S2. For the small clusters, we list the gene symbols in the most significant subcategory.
(0.12 MB PDF)

Table S3 **Putative BMAL1 targets with transcription factor activity (DAVID, GO:003700).** Additional columns include the rank of BMAL1 binding strength, the *p*-value for cyclic mRNA expression (data as in Figure 6; significant values, *p<*0.05, are in bold), and phase of mRNA expression. For the nuclear receptors, we also indicate results for mRNA expression patterns by real-time PCR in mouse liver [27]. According to those analyses, all 18 bound receptors are expressed and 9/18 show circadian accumulation.
(0.12 MB PDF)

Table S4 **Enriched KEGG pathways identified with DAVID (p<0.05).** The BMAL1 putative targets in the three most significant pathways are shown in Figure S5.
(0.23 MB PDF)

Table S5 **PSWM for the E1-E2 motif.** E1 goes from position 1 to 13, position 14 corresponds to the spacer, and E2 goes from position 15 to 27.
(0.05 MB PDF)

Table S6 **TaqMan probes for ChIP-PCR measurements.**
(0.04 MB PDF)

Table S7 **TaqMan probes for mRNA measurements.**
(0.05 MB PDF)

Table S8 **Annealing primers for EMSA.**
(0.04 MB PDF)

Table S9 **Annealing primers for transactivation assays.**
(0.05 MB PDF)

Text S1 **Supplementary methods.**
(0.09 MB PDF)

Text S2 **List of all BMAL1 sites with annotations and binding strength at each time point.**
(0.20 MB TXT)

Text S3 **List of BMAL1 sites near RCGs.**
(0.01 MB TXT)

## Abbreviations

bHLH: basic helix-loop-helix
ChIP: chromatin immunoprecipitation
ChIP-Seq: chromatin immunoprecipitation combined with deep sequencing
EMSA: electromobility shift assay
HMM: hidden Markov model
KEGG: Kyoto Encyclopedia of Genes and Genomes
qPCR: quantitative PCR
PSWM: position-specific weight matrix
RCG: reference clock gene
TSS: transcription start site
ZT: Zeitgeber time
