# Supplementary figures and images for: Genome-Wide and Phase-Specific DNA-Binding Rhythms of BMAL1 Control Circadian Output Functions in Mouse Liver
Source: PLoS Biol. 2011 Feb 22;9(2):e1000595. doi: 10.1371/journal.pbio.1000595 (PMC3043000; doi:10.1371/journal.pbio.1000595)

**A**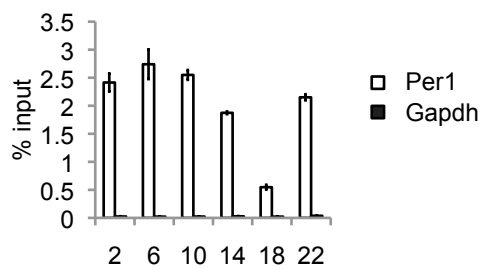**B**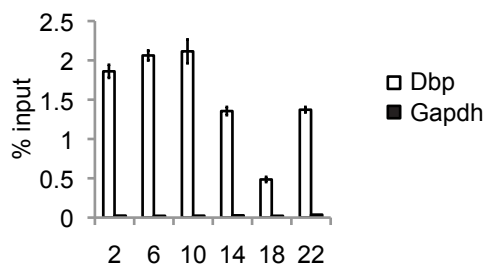

Supplement: Figure S1 — BMAL1 ChIP-qPCR at control loci. Two positive control loci, the Per1 promoter (A) and the Dbp site in intron 2 (B), show circadian BMAL1 binding. Fold enrichments relative to glyceraldehyde 3-phosphate dehydrogenase (Gapdh) are greater than 100-fold at ZT6 and about 10-fold at ZT18. (0.33 MB PDF) [file pbio.1000595.s001.pdf]

A

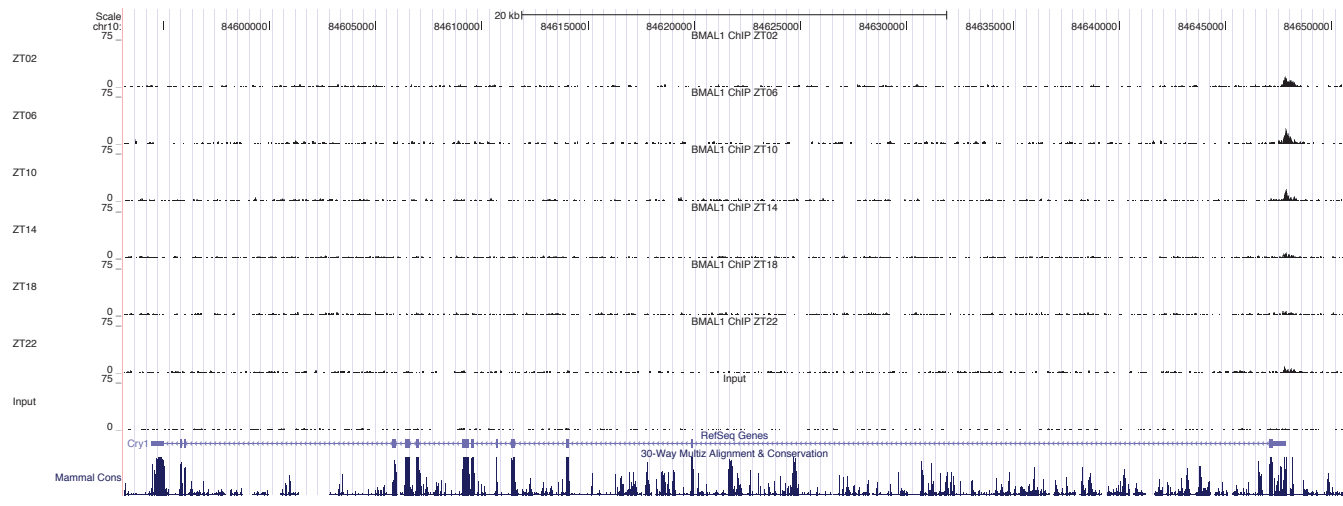

B

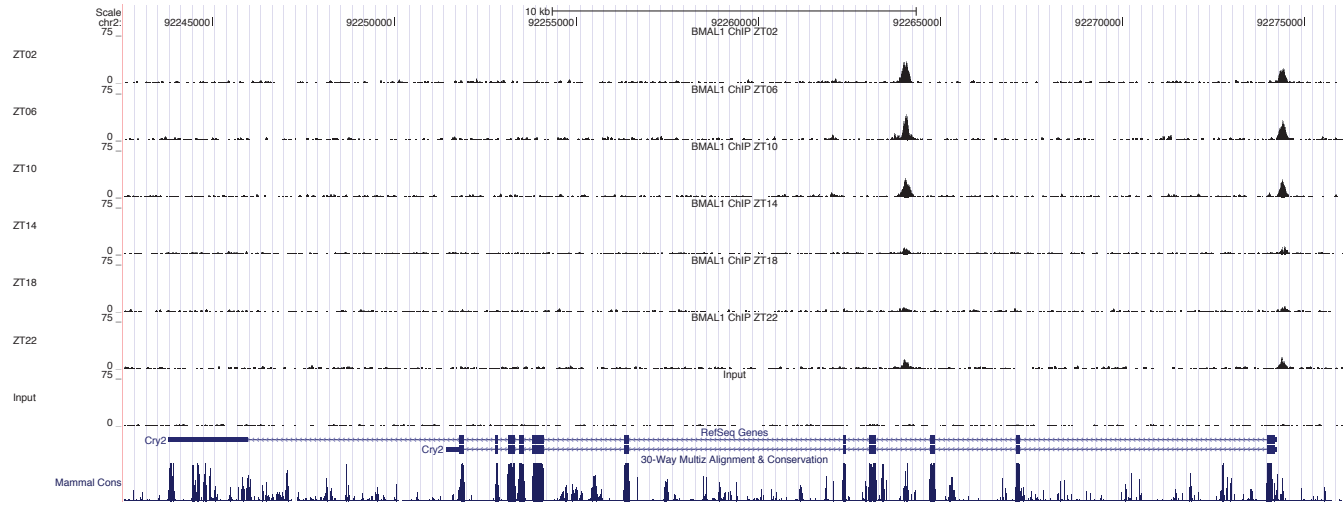

C

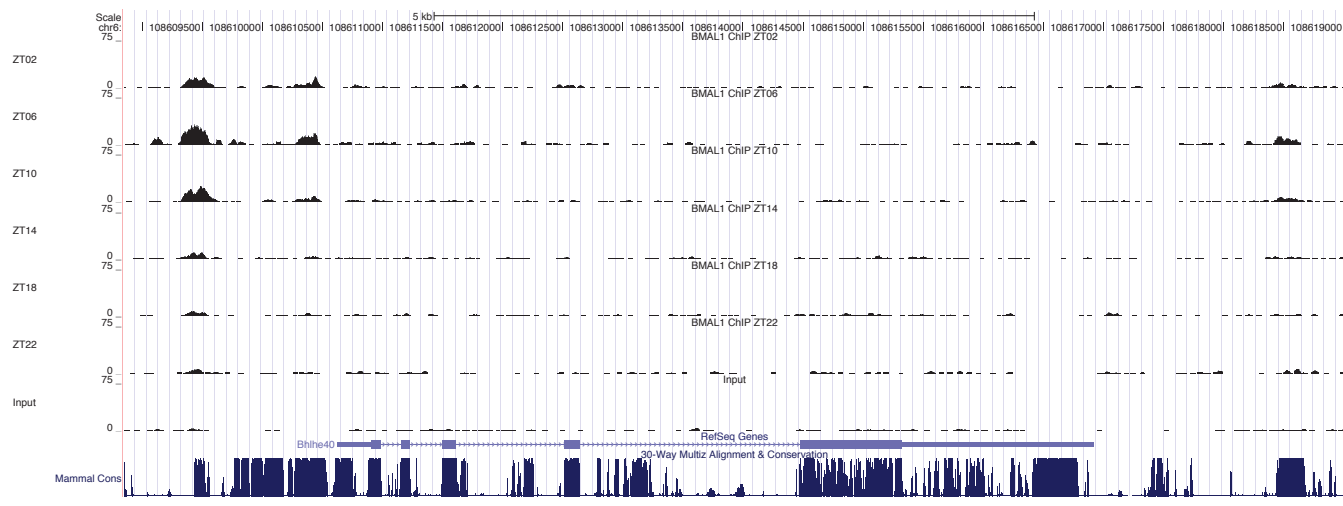

D

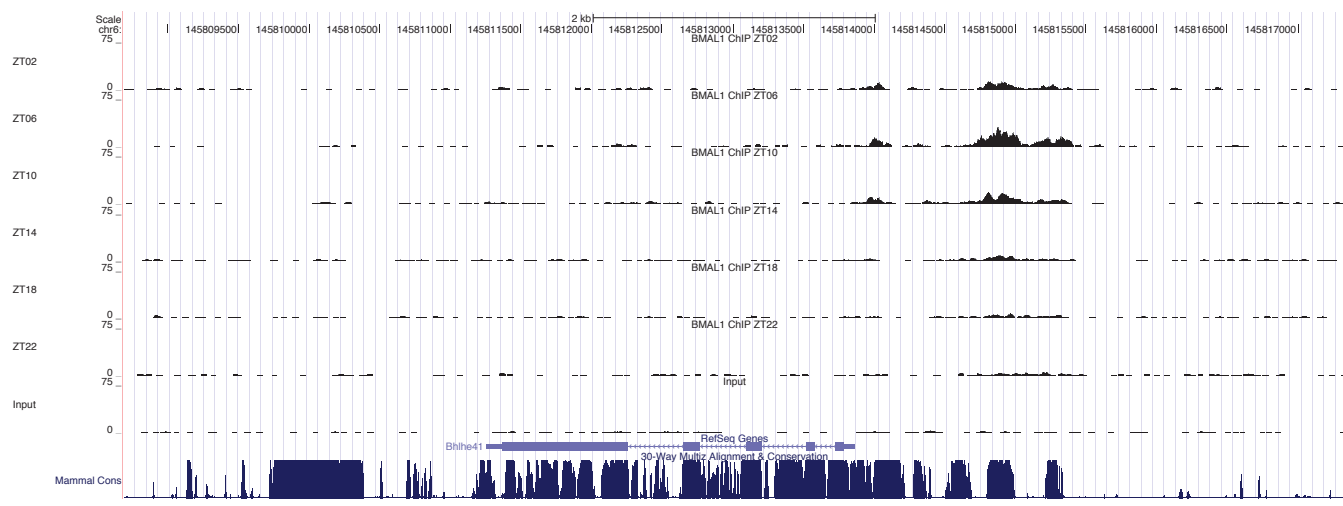

E

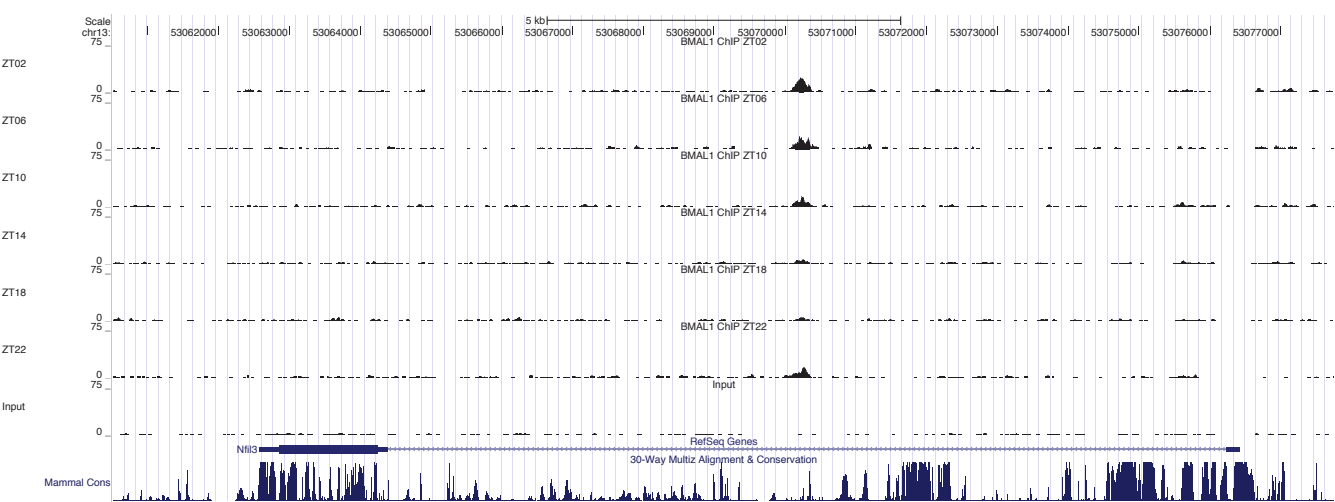

F

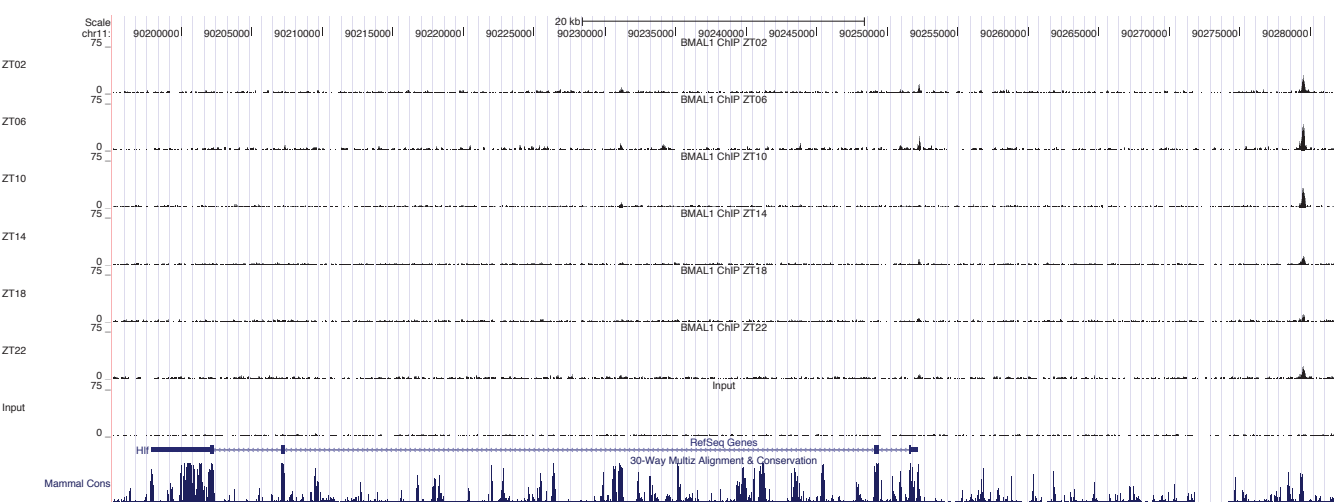

G

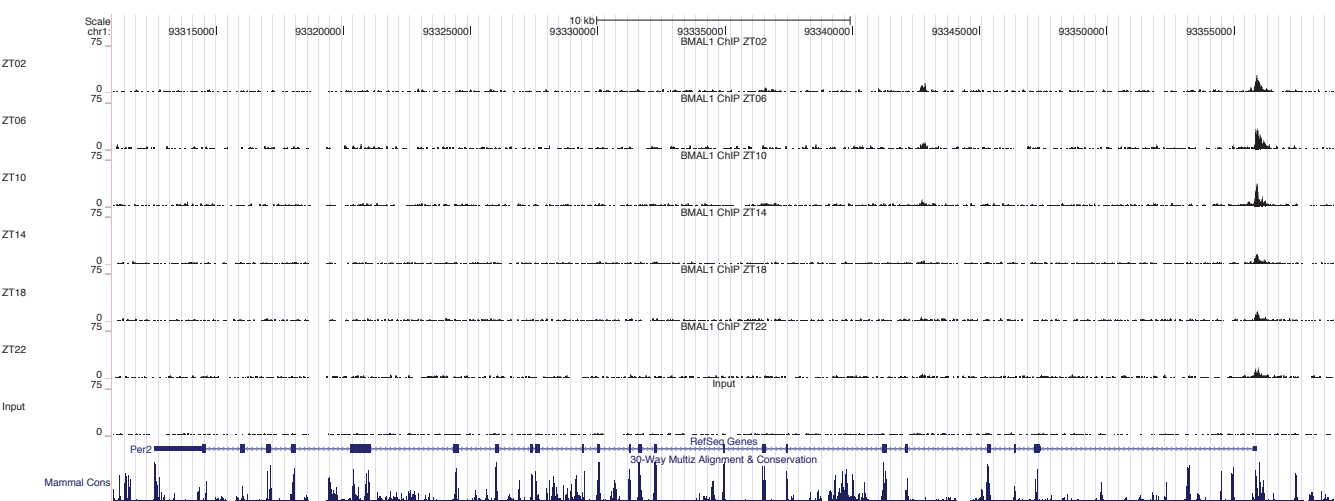

H

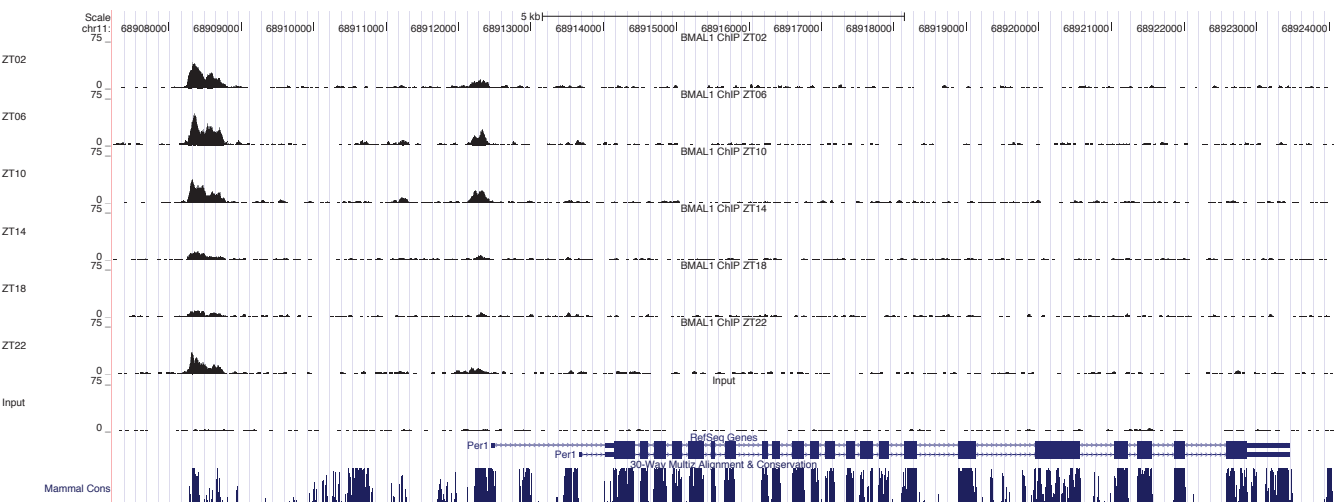

**I**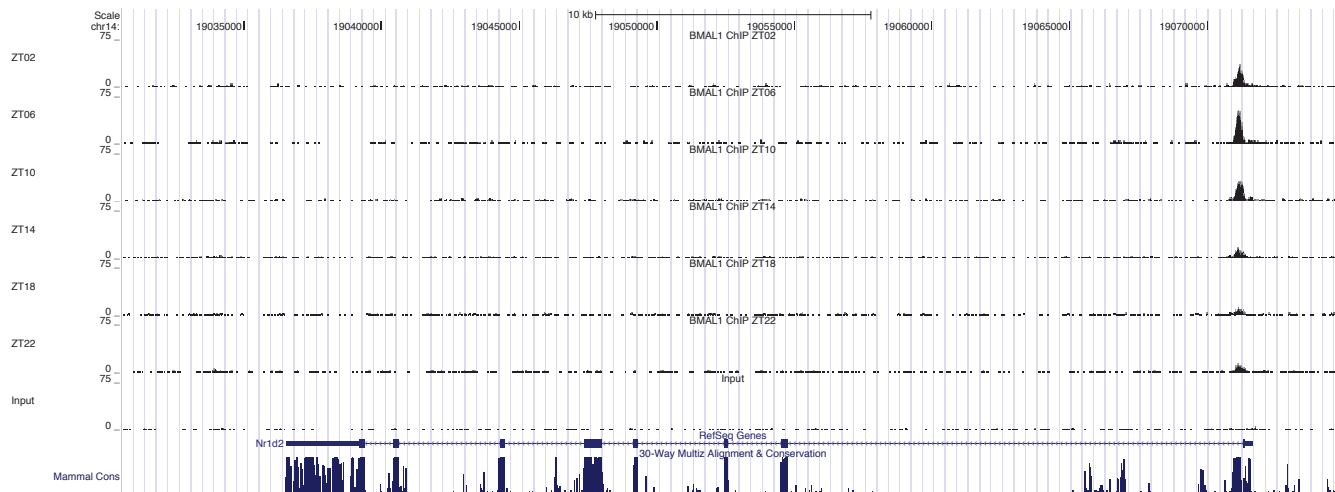**J**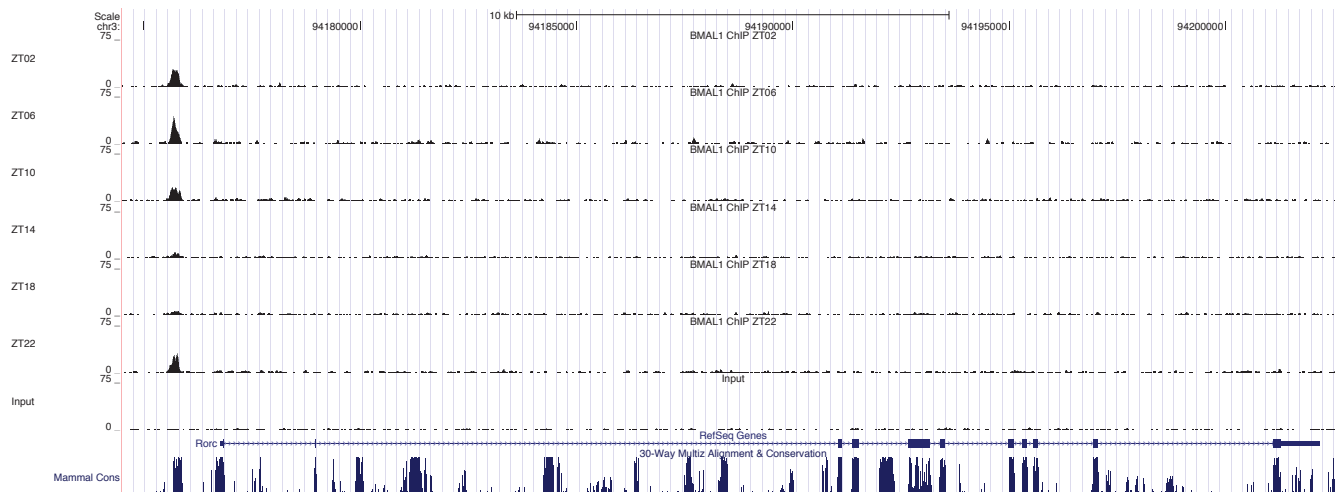**K**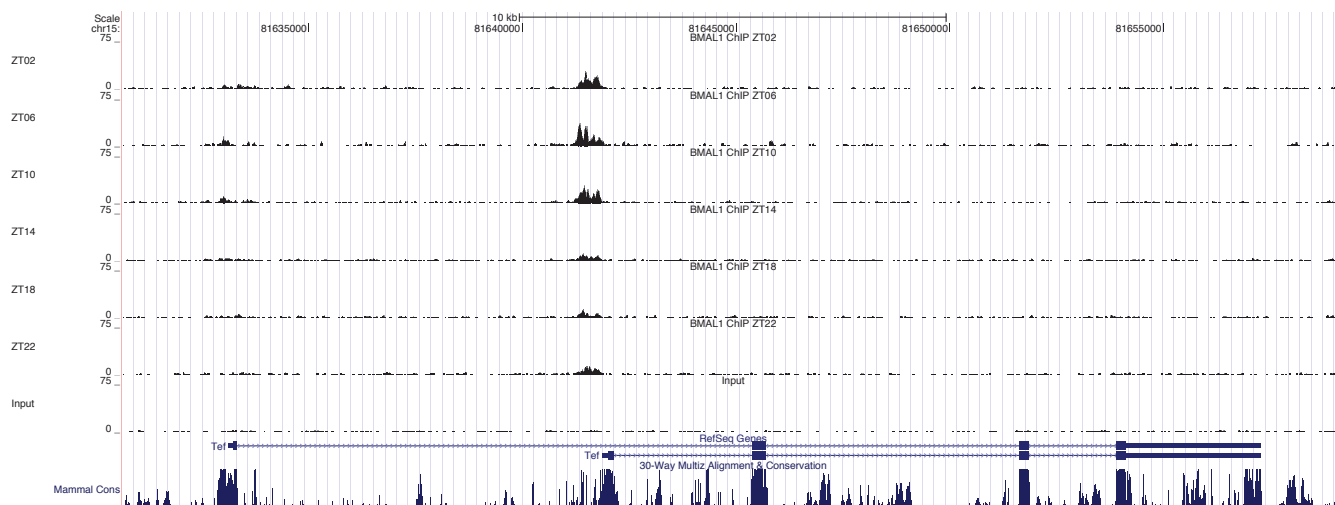

Supplement: Figure S2 — ChIP-Seq time series at circadian reference genes. Data viewed in the UCSC Genome Browser showing two BMAL1 sites in Cry1 (A), two in Cry2 (B), two in Dec1 (C), four in Dec2 (D), three in E4bp4 (E), three in Hlf (F), five in Per1 (G), two in Per2 (H), one in Rev-Erbβ (I), one in Rorγ (J), and four in Tef (K) loci. RefSeq annotation and PhastCons placental mammal conservation score are displayed. (2.60 MB PDF) [file pbio.1000595.s002.pdf]

**A**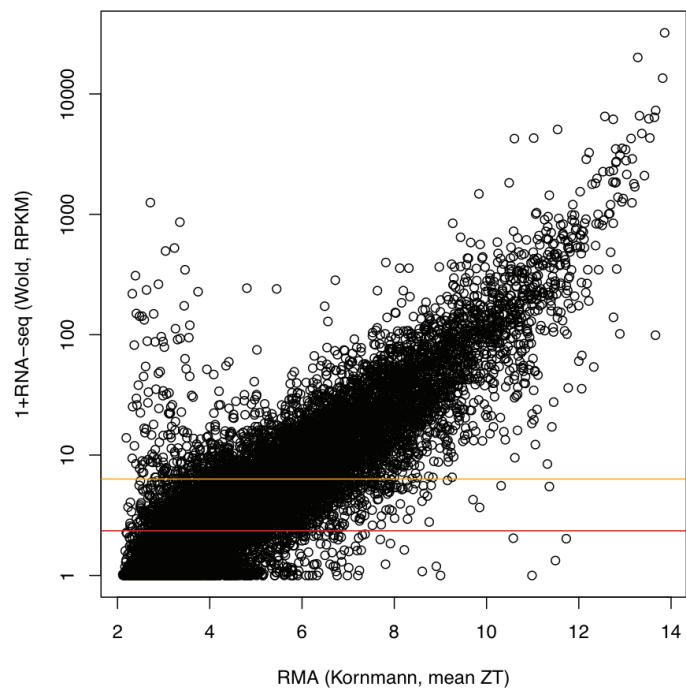**B**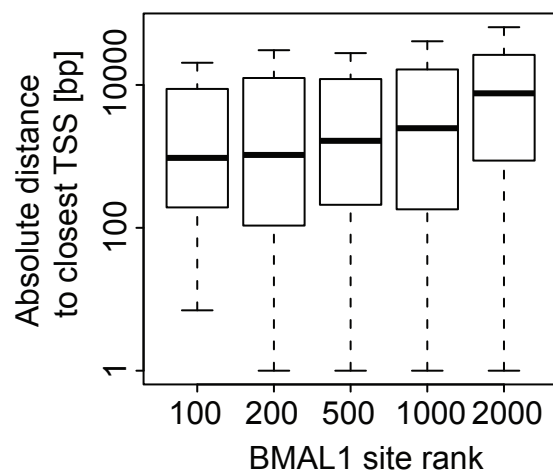

Supplement: Figure S3 — Liver RNA-Seq data define the liver-specific transcriptome, and binding strength depends on distance to genes. (A) Defining the liver-specific transcriptome from RNA-Seq data. Number of reads per kilobase per million mapped reads (RPKM) from RNA-Seq data [41] correlates with microarray data (normalized with RMA) averaged over time points from [31]. Liver-expressed genes were defined as genes with more than 1.35 reads per kilobase per million mapped reads (red line, 50% percentile). (B) Stronger BMAL1 sites are located closer to TSSs than weaker sites. The sites are binned according to rank, as in Figure 2E. (0.94 MB PDF) [file pbio.1000595.s003.pdf]

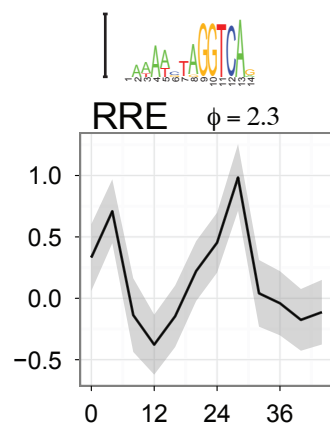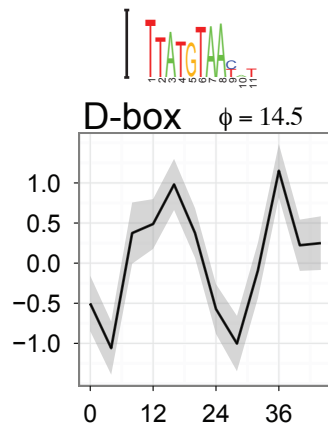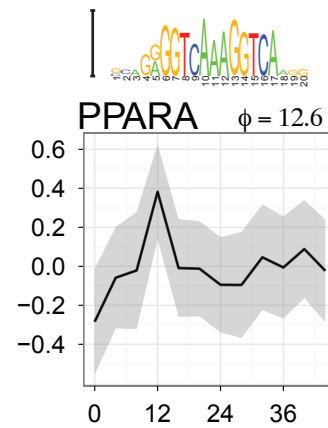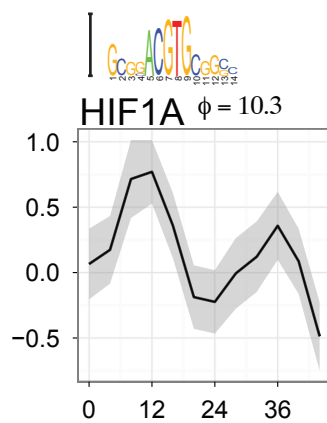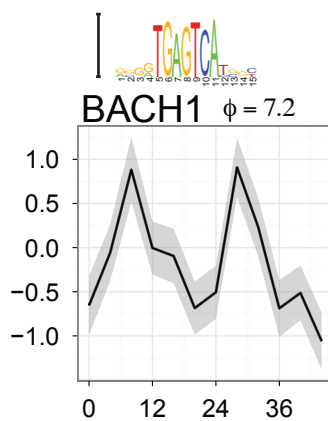

Supplement: Figure S4 — Inferring transcription factor activities from linear regression models. Inferred activity profiles (Amt) for BMAL1 targets (see Materials and Methods). Microarray data from [31] were used together with the PSWMs of the corresponding transcription factors (SwissRegulon database) to infer motif activities. Logo of the PSWM is shown above each profile, and phase of peak activity is indicated. Grey shades represent the standard errors of the linear regression at each time point. Only profiles with cyclic activity profiles are shown (Fisher test, p<0.05; see Materials and Methods). RRE stands for ROR response element, D-box is the DBP consensus element, PPARα is the Pparα binding site, HIF1A is a bHLH regulator, and BACH1 is a CNC-bZip leucine zipper protein. (0.50 MB PDF) [file pbio.1000595.s004.pdf]

A

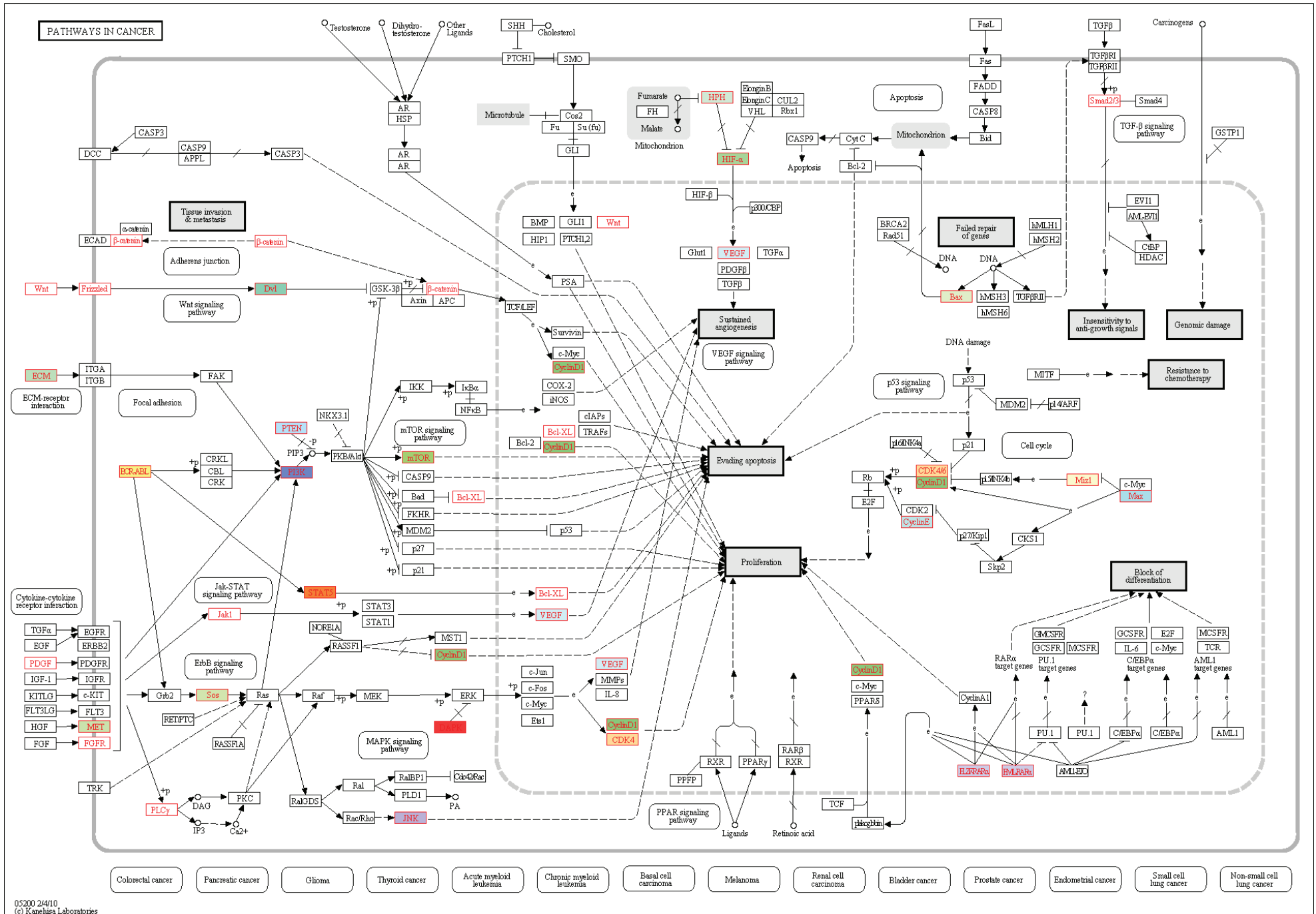

B

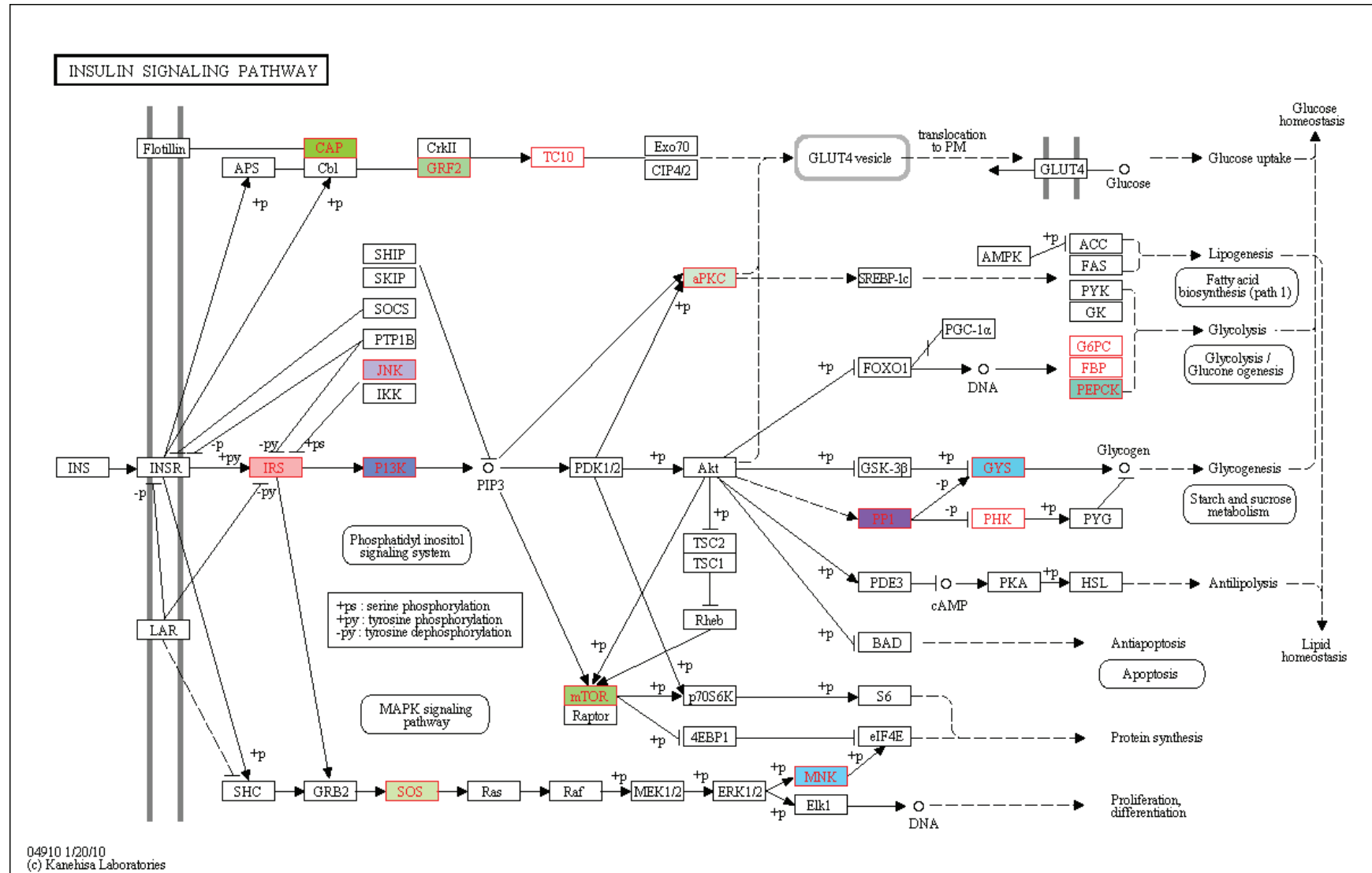

C

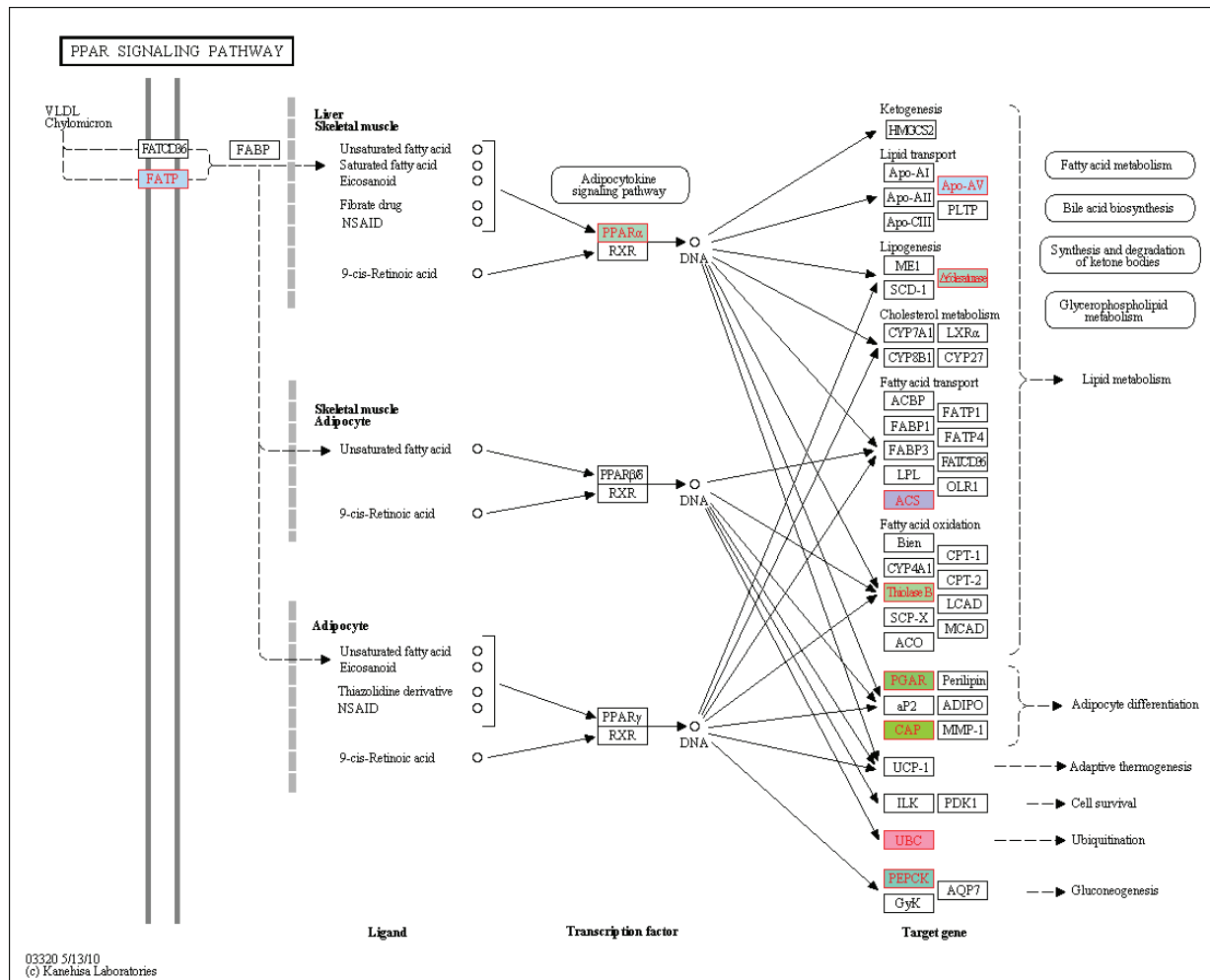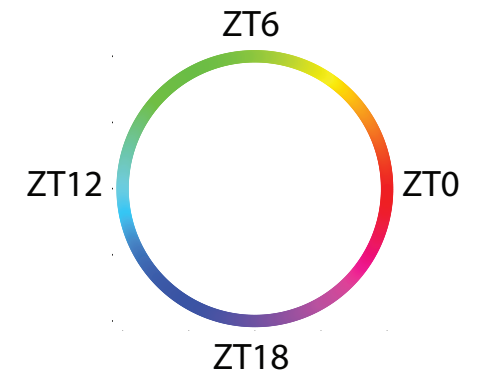

Supplement: Figure S5 — BMAL1 targets in KEGG pathways. (A) BMAL1 targets in the “Pathways in Cancer” KEGG pathway. Targets are colored according to phase of their mRNA expression in the data from [31]. The color legend is given in (C). All targets are shown as red boxes, but only those with well-defined phases (F24>0.2) are colored. (B) BMAL1 targets in the insulin signaling pathway. (C) BMAL1 targets in the Pparα signaling pathway. These graphs were generated using KEGG Mapper (http://www.genome.jp/kegg/tool/color_pathway.html). (1.31 MB PDF) [file pbio.1000595.s005.pdf]

A

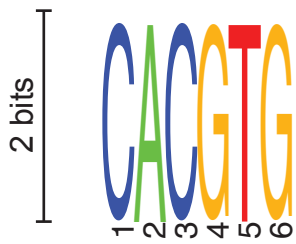

B

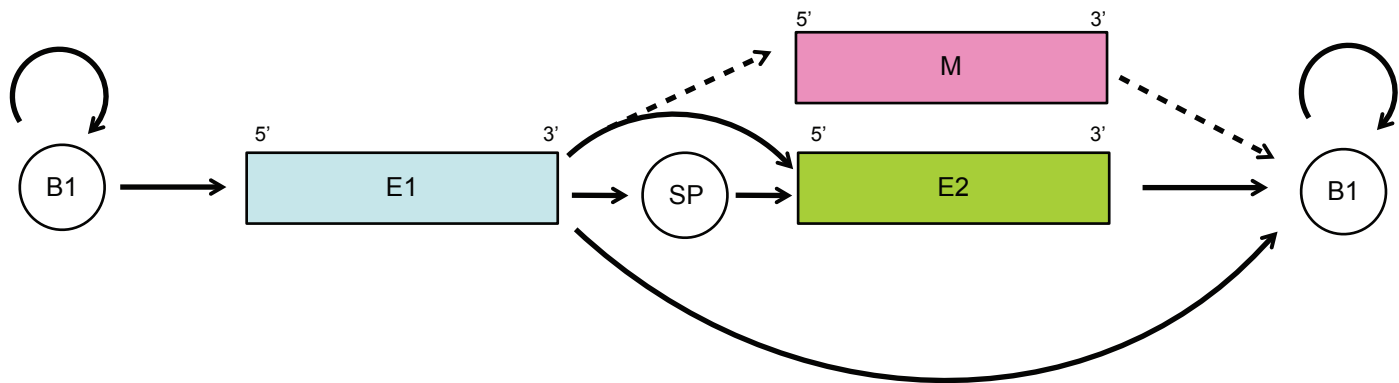

Supplement: Figure S6 — Weight matrix and structure of the HMM used for sequence analysis. (A) Logo of the E-box PSWM used for autocorrelation analysis. At each position of the PSWM, the most probable letter has p = 0.96875, while the others have p = 0.03125. (B) Structure of the HMM. E1 and E2 model, respectively, the collection of hidden states of the first and second E-box. M states allow for filtering of spurious signal, namely GTGT repeats. B1 and SP represent, respectively, background and spacer states. For simplicity, the reverse complement of the HMM is not shown here. (0.35 MB PDF) [file pbio.1000595.s006.pdf]

**A**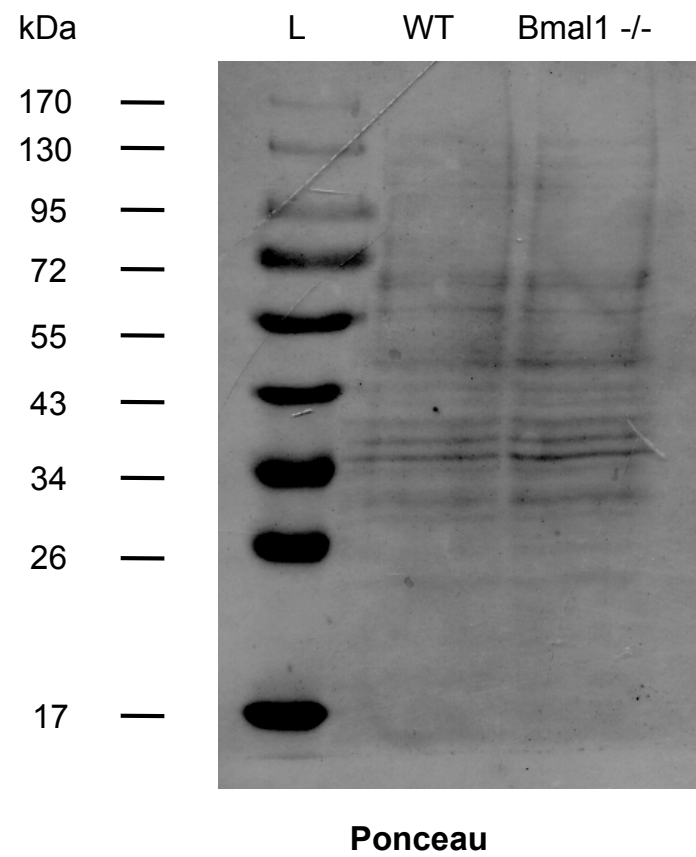**B**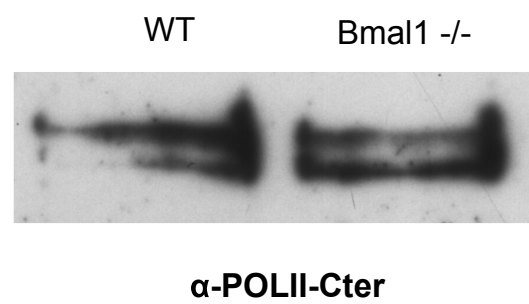**C**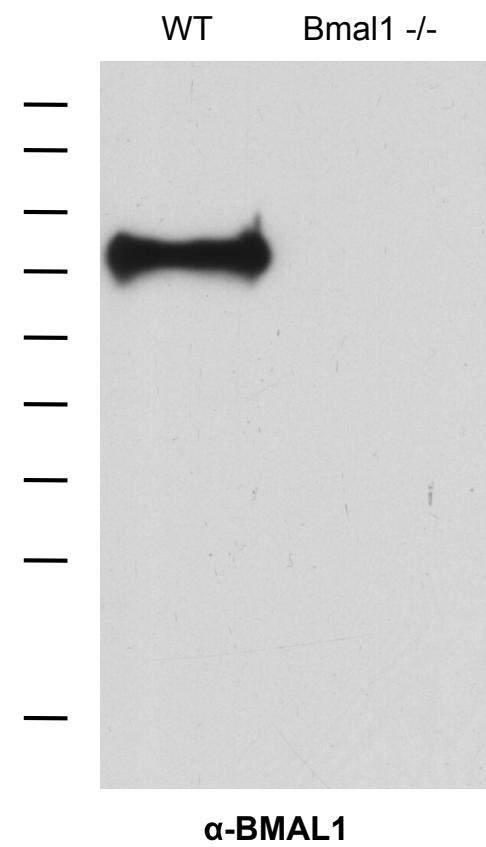

Supplement: Figure S8 — The anti-BMAL1 antibody recognizes specifically BMAL1. Ponceau staining (A) and Western blot (B and C) of nuclear extracts (15 ug) from wild-type and Bmal1 −/− mouse liver at ZT6. The nuclear extracts were electrophoresed on a 12% SDS-PAGE gel, transferred onto a nitrocellulose membrane, and detected using anti-POLII Cter (ab817-100, Abcam) (B) and anti-BMAL1 antibodies (C). The sequence of the peptide used for the immunization is located at the C-terminal of the mouse BMAL1 protein: LEADAGLGGPVDFSDLPWPL. (3.48 MB PDF) [file pbio.1000595.s008.pdf]
