# Supplementary material for: Genome-Wide and Phase-Specific DNA-Binding Rhythms of BMAL1 Control Circadian Output Functions in Mouse Liver
Source: PLoS Biol. 2011 Feb 22;9(2):e1000595. doi: 10.1371/journal.pbio.1000595 (PMC3043000; doi:10.1371/journal.pbio.1000595)
Supplement: Figure S7 — Pre-mRNA and mRNA measurements of longer lived transcripts. (A) mRNA transcript stability may explain lag and relative amplitude between pre-mRNA and mRNA accumulation in the Gys2, March8, and Qdpr transcripts. Experiments were performed as described in Figure 7A–7C. Approximate half-lives for March8 and Qdpr are 5.4 h and >10 h, while that for Gys2 is not available (see [B]). (B) mRNA half-lives from mouse embryonic stem cells [61] and mouse fibroblasts [62] for the genes in Figure 7A–7C and (A). When several measurements from the same cell line were available, we took the mean. (0.51 MB PDF) [file pbio.1000595.s007.pdf]

A

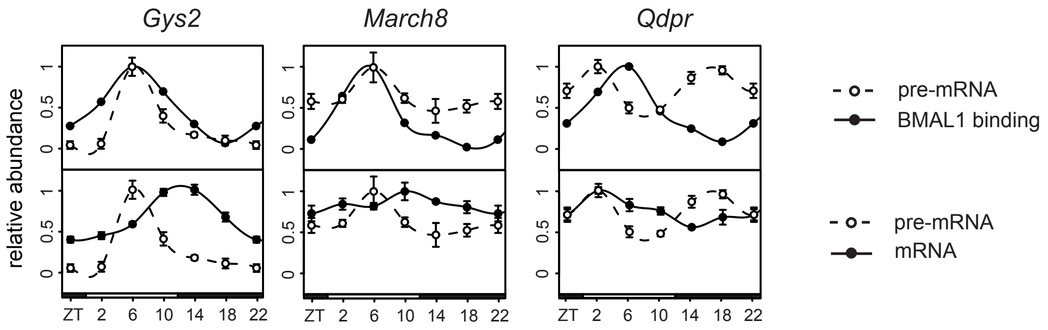

B

Gene Name	mRNA half-life [hours]	
	Sharova et al.	Friedel et al.
Dec2	3.3	-
Cry1	3.6	3.9
Cry2	4.2	-
Dbp	5.0	-
March8	5.4	-
Rev-Erbα	3.1	-
Rev-Erbβ	4.3	3.0
Per1	3.7	-
Per2	2.9	0.9
Qdpr	9.4	34.8
Rorγ	5.0	-
Tef	7.1	3.7
