# Supplementary material for: Genome-Wide and Phase-Specific DNA-Binding Rhythms of BMAL1 Control Circadian Output Functions in Mouse Liver
Source: PLoS Biol. 2011 Feb 22;9(2):e1000595. doi: 10.1371/journal.pbio.1000595 (PMC3043000; doi:10.1371/journal.pbio.1000595)
Supplement: Table S1 — Sequencing data: number of sequenced and non-redundant tags at each time point. (0.05 MB PDF) [file pbio.1000595.s009.pdf]

Table S1. Sequencing data: number of sequenced and non-redundant tags at each timepoint.

	Input	ZT2	ZT6	ZT10	ZT14	ZT18	ZT22
# tags mapped [in millions]	39.3	56.8	71.5	54.4	74.4	93.2	54.7
# non-redundant unique tags [in millions]	18	24.5	18.8	26.8	31.3	26.6	25.1
# tag number in all 2049 sites	10090	60391	77438	57551	30025	10927	30786
