# Supplementary material for: Genome-Wide and Phase-Specific DNA-Binding Rhythms of BMAL1 Control Circadian Output Functions in Mouse Liver
Source: PLoS Biol. 2011 Feb 22;9(2):e1000595. doi: 10.1371/journal.pbio.1000595 (PMC3043000; doi:10.1371/journal.pbio.1000595)
Supplement: Table S2 — Functional annotation clustering of putative BMAL1 targets using DAVID tools. These annotations link the sites to the closest gene irrespective of the distance. In total, 1,551 out of 2,049 sites have a functional annotation. For details regarding the positions and binding strength of these sites, see Text S2. For the small clusters, we list the gene symbols in the most significant subcategory. (0.12 MB PDF) [file pbio.1000595.s010.pdf]

Table S2. Functional Annotation Clustering of putative BMAL1 targets using DAVID tools.

**Cluster 1**	**Enrichment Score: 7.570281784314562**			
Category	Term	#	P	B
INTERPRO	IPR001723:Steroid hormone receptor	18	3.51E-09	5.82E-06
INTERPRO	IPR008946:Nuclear hormone receptor, ligand-binding	18	5.06E-09	4.18E-06
INTERPRO	IPR000536:Nuclear hormone receptor, ligand-binding, core	18	5.06E-09	4.18E-06
GOTERM_MF_FAT	GO:0003707~steroid hormone receptor activity	18	1.19E-08	1.18E-05
SMART	SM00430:HOLI	18	1.47E-08	4.43E-06
Esrra, Esrrg, Hnf4a, Hnf4g, Nr0b2, Nr1d1, Nr1d2, Nr1h4, Nr1i2, Nr2f2, Nr3c2, Nr5a2, Ppara, Rara, Rarb, Rora, Rorc, Thra				
**Cluster 2**	**Enrichment Score: 5.385647810701173**			
Category	Term	#	P	B
GOTERM_BP_FAT	GO:0006641~triglyceride metabolic process	13	2.02E-06	9.87E-04
GOTERM_BP_FAT	GO:0006639~acylglycerol metabolic process	14	2.64E-06	1.11E-03
GOTERM_BP_FAT	GO:0006662~glycerol ether metabolic process	14	4.66E-06	1.52E-03
GOTERM_BP_FAT	GO:0006638~neutral lipid metabolic process	14	4.66E-06	1.52E-03
GOTERM_BP_FAT	GO:0018904~organic ether metabolic process	14	1.02E-05	2.14E-03
Agpat6, Apoa5, Dgat2, G6pc, Insig1, Insig2, Mogat2, Pck1, Pcsk9, Pnpla2, Pnpla3, Sirt1, Slc22a4				
**Cluster 3**	**Enrichment Score: 5.076823004927006**			
Category	Term	#	P	B
SP_PIR_KEYWORDS	transcription regulation	156	6.36E-08	1.46E-05
SP_PIR_KEYWORDS	Transcription	161	1.91E-05	9.73E-04
GOTERM_BP_FAT	GO:0045449~regulation of transcription	194	3.59E-05	5.54E-03
GOTERM_BP_FAT	GO:0006350~transcription	157	1.13E-04	1.10E-02
**Cluster 4**	**Enrichment Score: 4.826858791167763**			
Category	Term	#	P	B
GOTERM_BP_FAT	GO:0016481~negative regulation of transcription	51	1.52E-06	1.11E-03
GOTERM_BP_FAT	GO:0045934~negative regulation of nucleobase, …	52	4.53E-06	1.66E-03
GOTERM_BP_FAT	GO:0010629~negative regulation of gene expression	53	5.32E-06	1.56E-03
GOTERM_BP_FAT	GO:0051172~negative regulation of nitrogen compound …	52	6.06E-06	1.48E-03
GOTERM_BP_FAT	GO:0031327~negative regulation of cellular biosynthetic process	54	9.87E-06	2.23E-03
**Cluster 5**	**Enrichment Score: 4.579854703068408**			
Category	Term	#	P	B
INTERPRO	IPR004827:Basic-leucine zipper (bZIP) transcription factor	15	6.02E-06	1.99E-03
SMART	SM00338:BRLZ	15	1.38E-05	1.39E-03
UP_SEQ_FEATURE	domain:Leucine-zipper	20	2.19E-04	8.71E-02
**Cluster 6**	**Enrichment Score: 4.172511153755323**			
Category	Term	#	P	B
SP_PIR_KEYWORDS	nucleotide-binding	154	4.33E-06	3.96E-04
SP_PIR_KEYWORDS	atp-binding	126	6.73E-06	3.85E-04
GOTERM_MF_FAT	GO:0000166~nucleotide binding	196	3.49E-05	8.60E-03
GOTERM_MF_FAT	GO:0005524~ATP binding	137	6.36E-05	1.25E-02
GOTERM_MF_FAT	GO:0030554~adenyl nucleotide binding	143	1.04E-04	1.45E-02

**Cluster 7**	**Enrichment Score: 3.970366701916962**			
Category	Term	#	P	B
GOTERM_BP_FAT	GO:0045893~positive regulation of transcription, DNA-dependent	52	1.69E-05	3.11E-03
GOTERM_BP_FAT	GO:0051254~positive regulation of RNA metabolic process	52	2.06E-05	3.36E-03
GOTERM_BP_FAT	GO:0045941~positive regulation of transcription	56	4.06E-05	5.95E-03
GOTERM_BP_FAT	GO:0010628~positive regulation of gene expression	57	4.46E-05	5.69E-03
GOTERM_BP_FAT	GO:0010604~positive regulation of macromolecule metabolic …	68	1.04E-04	1.05E-02
**Cluster 8**	**Enrichment Score: 3.683169135128673**			
Category	Term	#	P	B
GOTERM_BP_FAT	GO:0005996~monosaccharide metabolic process	29	6.67E-05	7.50E-03
GOTERM_BP_FAT	GO:0019318~hexose metabolic process	25	3.50E-04	2.67E-02
GOTERM_BP_FAT	GO:0006006~glucose metabolic process	22	3.82E-04	2.84E-02
Aldoart2, Aldob, B4galt1, Car5a, Csgalnact1, Fbp1, G6pc, G6pc3, Gbe1, Gm10481, Gne, Gnpnat1, Gys2, Hibadh, Il6st, Ldha, Man2a1, Pck1, Pcx, Pdk1, Pfkfb3, Pfkl, Pgm2l1, Phkb, Ppara, Ppp1r3b, Ppp1r3c, Ugt1a10, Wdtc1				
**Cluster 9**	**Enrichment Score: 3.261796532650017**			
Category	Term	#	PValue	B
INTERPRO	IPR001849:Pleckstrin homology	34	1.80E-04	4.85E-02
SMART	SM00233:PH	34	6.37E-04	4.70E-02
UP_SEQ_FEATURE	domain:PH	28	1.43E-03	4.05E-01
**Cluster 10**	**Enrichment Score: 2.760908956897479**			
Category	Term	#	P	B
UP_SEQ_FEATURE	zinc finger region:PHD-type 1	11	1.47E-05	2.12E-02
UP_SEQ_FEATURE	zinc finger region:PHD-type 2	10	3.69E-05	3.52E-02
INTERPRO	IPR019787:Zinc finger, PHD-finger	13	5.81E-03	4.53E-01
INTERPRO	IPR001965:Zinc finger, PHD-type	13	1.44E-02	6.63E-01
SMART	SM00249:PHD	13	2.42E-02	4.90E-01
**Cluster 11**	**Enrichment Score: 2.6581652848216746**			
Category	Term	#	P	B
GOTERM_BP_FAT	GO:0046463~acylglycerol biosynthetic process	6	6.95E-04	4.75E-02
GOTERM_BP_FAT	GO:0046460~neutral lipid biosynthetic process	6	6.95E-04	4.75E-02
GOTERM_BP_FAT	GO:0046504~glycerol ether biosynthetic process	6	1.07E-03	6.33E-02
GOTERM_BP_FAT	GO:0019432~triglyceride biosynthetic process	5	1.11E-03	6.30E-02
GOTERM_BP_FAT	GO:0045017~glycerolipid biosynthetic process	8	8.97E-02	7.41E-01
Agpat6, Dgat2, Mogat1, Mogat2, Pck1, Pnpla3				
**Cluster 12**	**Enrichment Score: 2.574218919122059**			
Category	Term	#	P	B
GOTERM_MF_FAT	GO:0043169~cation binding	307	2.12E-03	1.10E-01
GOTERM_MF_FAT	GO:0043167~ion binding	310	2.38E-03	1.17E-01
GOTERM_MF_FAT	GO:0046872~metal ion binding	302	3.76E-03	1.56E-01
**Cluster 13**	**Enrichment Score: 2.4615864721249996**			
Category	Term	#	P	B

GOTERM_BP_FAT	GO:0006094~gluconeogenesis	7	1.49E-03	8.08E-02
GOTERM_BP_FAT	GO:0046364~monosaccharide biosynthetic process	8	3.66E-03	1.51E-01
GOTERM_BP_FAT	GO:0006090~pyruvate metabolic process	7	5.11E-03	1.89E-01
GOTERM_BP_FAT	GO:0019319~hexose biosynthetic process	7	5.11E-03	1.89E-01
Aldob, Car5a, Fbp1, G6pc, G6pc3, Pck1, Pcx				
**Cluster 14**	**Enrichment Score: 2.374480605027989**			
Category	Term	#	P	B
INTERPRO	IPR001610:PAC motif	8	1.05E-03	1.75E-01
SMART	SM00086:PAC	8	1.62E-03	7.82E-02
INTERPRO	IPR000014:PAS	8	3.80E-03	3.84E-01
INTERPRO	IPR013655:PAS fold-3	6	4.78E-03	4.33E-01
SMART	SM00091:PAS	8	5.73E-03	2.20E-01
**Cluster 15**	**Enrichment Score: 2.328696704988734**			
Category	Term	#	P	B
GOTERM_BP_FAT	GO:0048514~blood vessel morphogenesis	25	3.24E-03	1.43E-01
GOTERM_BP_FAT	GO:0001944~vasculature development	29	4.85E-03	1.82E-01
GOTERM_BP_FAT	GO:0001568~blood vessel development	28	6.57E-03	2.28E-01
**Cluster 16**	**Enrichment Score: 2.2269434033112434**			
Category	Term	#	P	B
GOTERM_BP_FAT	GO:0042304~regulation of fatty acid biosynthetic process	5	1.89E-03	9.59E-02
GOTERM_BP_FAT	GO:0019217~regulation of fatty acid metabolic process	6	6.90E-03	2.32E-01
GOTERM_BP_FAT	GO:0010565~regulation of cellular ketone metabolic process	6	1.60E-02	3.48E-01
Hnf4a, Insig1, Insig2, Scap, Wdtc1				
**Cluster 17**	**Enrichment Score: 2.02601777896621**			
Category	Term	#	P	B
INTERPRO	IPR001841:Zinc finger, RING-type	32	3.49E-03	3.83E-01
SMART	SM00184:RING	32	9.69E-03	3.08E-01
INTERPRO	IPR017907:Zinc finger, RING-type, conserved site	26	2.47E-02	7.60E-01
**Cluster 18**	**Enrichment Score: 2.012102589102119**			
Category	Term	#	P	B
GOTERM_BP_FAT	GO:0006468~protein amino acid phosphorylation	60	7.08E-03	2.32E-01
GOTERM_BP_FAT	GO:0016310~phosphorylation	65	1.02E-02	2.82E-01
GOTERM_BP_FAT	GO:0006793~phosphorus metabolic process	76	1.11E-02	2.95E-01
GOTERM_BP_FAT	GO:0006796~phosphate metabolic process	76	1.11E-02	2.95E-01

The annotations link the sites to the closest gene irrespective of the distance. In total, 1551 out of 2049 sites have a functional annotation. For details regarding the positions and strength of these sites, we refer to the supplemental file BMAL1_sites.txt. For the small clusters, we list the genes symbols in the most significant subcategory.[1]P = p-value, B = Benjamini corrected p-value.
