# Supplementary material for: Genome-Wide and Phase-Specific DNA-Binding Rhythms of BMAL1 Control Circadian Output Functions in Mouse Liver
Source: PLoS Biol. 2011 Feb 22;9(2):e1000595. doi: 10.1371/journal.pbio.1000595 (PMC3043000; doi:10.1371/journal.pbio.1000595)
Supplement: Table S3 — Putative BMAL1 targets with transcription factor activity (DAVID, GO:003700). Additional columns include the rank of BMAL1 binding strength, the p-value for cyclic mRNA expression (data as in Figure 6; significant values, p<0.05, are in bold), and phase of mRNA expression. For the nuclear receptors, we also indicate results for mRNA expression patterns by real-time PCR in mouse liver [27]. According to those analyses, all 18 bound receptors are expressed and 9/18 show circadian accumulation. (0.12 MB PDF) [file pbio.1000595.s011.pdf]

Table S3. Putative BMAL1 targets with transcription factor activity (DAVID, GO:003700).

Ensembl transcript ID	Symbol	Name	mRNA (qPCR)	rank	p-val	phase
		**Nuclear receptor**				
ENSMUST00000064941	Nr1d1	nuclear receptor subfamily 1, group D, member 1	Rhythmic	2	**0.0025**	5.9
ENSMUST00000143308	Nr1d2	nuclear receptor subfamily 1, group D, member 2	Rhythmic	5	**0.0044**	9.6
ENSMUST00000029795	Rorc	RAR-related orphan receptor gamma	Rhythmic	17	**0.0021**	19.7
ENSMUST00000126697	Nr3c2	nuclear receptor subfamily 3, group C, member 2	Non-Rhythmic	57	**0.0133**	7.0
ENSMUST00000108393	Hnf4g	hepatocyte nuclear factor 4, gamma	Non-Rhythmic	146	0.2121	5.6
ENSMUST00000113432	Esrra	estrogen related receptor, alpha	Rhythmic	228	0.1329	14.8
ENSMUST00000032768	Nr2f2	nuclear receptor subfamily 2, group F, member 2	Non-Rhythmic	296	**0.0298**	9.9
ENSMUST00000136453	Ppara	peroxisome proliferator activated receptor alpha	Rhythmic	302	0.0549	9.9
ENSMUST00000107473	Rara	retinoic acid receptor, alpha	Rhythmic	836	0.2703	16.3
ENSMUST00000042706	Nr0b2	nuclear receptor subfamily 0, group B, member 2	Rhythmic	1539	0.1962	23.8
ENSMUST00000023504	Nr1i2	nuclear receptor subfamily 1, group I, member 2	Non-Rhythmic	1591	0.1590	11.4
ENSMUST00000143507	Rora	RAR-related orphan receptor alpha	Non-Rhythmic	1655	0.0818	18.3
ENSMUST00000124072	Thra	thyroid hormone receptor alpha	Rhythmic	1694	**0.0052**	3.2
ENSMUST00000105297	Nr1h4	nuclear receptor subfamily 1, group H, member 4	Non-Rhythmic	1715	0.3493	13.0
ENSMUST00000051130	Hnf4a	hepatic nuclear factor 4, alpha	Non-Rhythmic	1746	**0.0070**	13.9
ENSMUST00000112609	Rarb	retinoic acid receptor, beta	Non-Rhythmic	1789	0.8635	6.3
ENSMUST00000127489	Esrrg	estrogen-related receptor gamma	Rhythmic	1942	0.6643	4.6
ENSMUST00000027649	Nr5a2	nuclear receptor subfamily 5, group A, member 2	Non-Rhythmic	1960	0.3627	23.8
		**Basic-leucine zipper (bZIP)**				
ENSMUST00000155439	Dbp	D site albumin promoter binding protein		4	**0.0013**	9.1
ENSMUST00000107883	Hlf	hepatic leukemia factor		10	**0.0035**	12.6
ENSMUST00000023024	Tef	thyrotroph embryonic factor		14	**0.0001**	11.4
ENSMUST00000071065	Nfil3	similar to NFIL3/E4BP4 transcription factor; nuclear factor, interleukin 3, regulated		55	**0.0004**	22.0
ENSMUST00000064922	Junb	Jun-B oncogene		60	0.0956	5.2
ENSMUST00000109104	Maf	similar to c-Maf long form		111	0.2219	11.0
ENSMUST00000152371	Nfe2l2	nuclear factor, erythroid derived 2, like 2		129	0.2674	12.3
ENSMUST00000108828	Atf7	activating transcription factor 7		471	0.6786	14.3
ENSMUST00000156958	Bach1	BTB and CNC homology 1		509	0.2342	10.0
ENSMUST00000099126	Mafb	v-maf musculoaponeurotic fibrosarcoma oncogene family, protein B (avian)		824	0.1211	12.0
ENSMUST00000151577	Mafk	v-maf musculoaponeurotic fibrosarcoma oncogene family, protein K (avian)		936	0.1959	6.5
ENSMUST00000015605	Atf6b	activating transcription factor 6 beta		965	0.2015	21.4
ENSMUST00000132438	Nfe2	nuclear factor, erythroid derived 2		1251	0.0522	1.4
ENSMUST00000048384	Creb3l3	cAMP responsive element binding protein 3-like 3		1501	0.0798	11.3
ENSMUST00000063084	Xbp1	X-box binding protein 1		1817	0.0883	16.8

**Basic helix-loop-helix**					
ENSMUST00000105345	Tcf3	transcription factor E2a	214	0.1032	3.8
ENSMUST00000112273	Epas1	endothelial PAS domain protein 1; similar to Endothelial PAS domain protein 1	390	0.2073	14.1
ENSMUST00000021530	Hif1a	hypoxia inducible factor 1, alpha subunit	407	0.0687	8.9
ENSMUST00000132150	Mnt	max binding protein	590	0.2315	22.4
ENSMUST00000084703	Arntl	aryl hydrocarbon receptor nuclear translocator-like	1406	**0.0005**	21.9
ENSMUST00000058636	Helt	Hey-like transcription factor (zebrafish)	1466	-	-
**Zinc finger**					
ENSMUST00000032174	Klf15	Kruppel-like factor 15	52	**0.0493**	11.2
ENSMUST00000038558	Klf16	Kruppel-like factor 16	185	0.1092	9.1
ENSMUST00000020982	Klf11	Kruppel-like factor 11	343	0.5339	5.7
ENSMUST00000028866	Zc3h8	zinc finger CCCH type containing 8	669	0.0624	12.3
ENSMUST00000098143	Nfx1	nuclear transcription factor, X-box binding 1	679	**0.0125**	7.8
ENSMUST00000006762	Snai3	snail homolog 3 (Drosophila)	780	0.8176	11.6
ENSMUST00000047734	Zfp281	zinc finger protein 281	1081	**0.0028**	10.9
ENSMUST00000034629	Hinfp	similar to MBD2-interacting zinc finger; histone H4 transcription factor	1092	0.5522	8.2
ENSMUST00000018491	Zkscan6	zinc finger with KRAB and SCAN domains 6	1564	0.7216	19.8
ENSMUST00000114667	Tshz1	teashirt zinc finger family member 1	1969	0.0947	12.6
**Homeobox**					
ENSMUST00000111429	Pou2f1	POU domain, class 2, transcription factor 1	512	0.0513	4.5
ENSMUST00000115145	Onecut2	one cut domain, family member 2	1506	0.0849	10.8
ENSMUST00000010319	Prox1	prospero-related homeobox 1	1761	0.2013	17.7
ENSMUST00000109693	Irx2	similar to iroquois-class homeobox protein IRX2	1801	0.7377	19.2
ENSMUST00000027878	Prrx1	paired related homeobox 1	1808	0.1990	18.0
ENSMUST00000137697	Satb1	special AT-rich sequence binding protein 1	2011	0.0429	3.3
ENSMUST00000077337	Irx1	Iroquois related homeobox 1 (Drosophila)	2037	0.2746	20.2
**SMAD domain**					
ENSMUST00000069557	Smad5	MAD homolog 5 (Drosophila)	601	0.0955	9.8
ENSMUST00000025453	Smad2	MAD homolog 2 (Drosophila)	901	0.4373	5.9
ENSMUST00000144323	Smad6	MAD homolog 6 (Drosophila)	1266	0.4842	20.8
ENSMUST00000066091	Smad1	MAD homolog 1 (Drosophila)	2031	0.3675	0.0
**Winged helix**					
ENSMUST00000105501	Foxo3	forkhead box O3	217	**0.0124**	10.6
ENSMUST00000111979	Pax2	paired box gene 2	361	-	-
ENSMUST00000106114	Foxk2	forkhead box K2	925	**0.0257**	10.8
ENSMUST00000079071	Tfdp1	transcription factor Dp 1; similar to Transcription factor Dp-1 (E2F dimerization partner 1)	975	0.1035	12.5
ENSMUST00000145869	Etv6	ets variant gene 6 (TEL oncogene)	993	0.4587	19.7
ENSMUST00000108920	Irf1	interferon regulatory factor 1	998	**0.0183**	2.7

ENSMUST00000034041	Irf2	interferon regulatory factor 2	1283	**0.0496**	10.3
ENSMUST00000140544	Ets2	E26 avian leukemia oncogene 2, 3' domain	1550	0.1107	6.8
**Others**					
ENSMUST00000131118	Arid3a	AT rich interactive domain 3A (BRIGHT-like)	116	0.6162	6.0
ENSMUST00000035101	Csrnp1	cysteine-serine-rich nuclear protein 1	216	**0.0400**	9.8
ENSMUST00000099070	Nfix	nuclear factor I/X	281	**0.0482**	9.3
ENSMUST00000112995	Tsc22d3	TSC22 domain family, member 3	337	**0.0169**	16.8
ENSMUST00000038107	Cited2	Cbp/p300-interacting transactivator, with Glu/Asp-rich carboxy-terminal domain, 2	427	0.4922	2.8
ENSMUST00000152145	Aff1	AF4/FMR2 family, member 1	810	**0.0363**	4.0
ENSMUST00000098937	Ecsit	ECSIT homolog (Drosophila)	865	0.3822	12.2
ENSMUST00000152082	Arap1	ArfGAP with RhoGAP domain, ankyrin repeat and PH domain 1	953	**0.0272**	2.9
ENSMUST00000130911	Nfyb	nuclear transcription factor-Y beta	1042	0.1259	6.2
ENSMUST00000078185	Nfic	nuclear factor I/C	1094	0.1632	16.4
ENSMUST00000079652	Sox7	SRY-box containing gene 7	1231	0.3954	10.3
ENSMUST00000107358	Stat5b	signal transducer and activator of transcription 5B	1313	**0.0020**	1.7
ENSMUST00000139138	Ctnnb1	catenin (cadherin associated protein), beta 1	1562	0.6917	21.9
ENSMUST00000072460	Mef2a	similar to Myocyte enhancer factor 2A; myocyte enhancer factor 2A	1689	0.2334	11.8

Additional columns include the rank of BMAL1 binding strength, the p-value for cyclic mRNA expression (data as in Fig. 6 significant values $p<0.05$ are in bold), and phase of mRNA expression given in hours. For the nuclear receptors, we also indicate results for mRNA expression patterns by real-time-PCR in mouse liver {Yang, 2006}. According to those all 18 bound receptors are expressed and 9/18 show circadian accumulation.
