# Supplementary material for: Genome-Wide and Phase-Specific DNA-Binding Rhythms of BMAL1 Control Circadian Output Functions in Mouse Liver
Source: PLoS Biol. 2011 Feb 22;9(2):e1000595. doi: 10.1371/journal.pbio.1000595 (PMC3043000; doi:10.1371/journal.pbio.1000595)
Supplement: Table S4 — Enriched KEGG pathways identified with DAVID (p<0.05). The BMAL1 putative targets in the three most significant pathways are shown in Figure S5. (0.23 MB PDF) [file pbio.1000595.s012.pdf]

Table S4. Enriched KEGG pathways identified with DAVID ($p<0.05$).

Category	Term	RT	Genes	Count	%	P-Value	Benjamini
KEGG_PATHWAY	Circadian rhythm	RT		9	0.6	4.0E-7	6.8E-5
KEGG_PATHWAY	Pathways in cancer	RT		39	2.7	5.0E-4	4.2E-2
KEGG_PATHWAY	Insulin signaling pathway	RT		18	1.2	1.1E-2	4.8E-1
KEGG_PATHWAY	Small cell lung cancer	RT		13	0.9	1.1E-2	3.9E-1
KEGG_PATHWAY	TGF-beta signaling pathway	RT		13	0.9	1.4E-2	3.8E-1
KEGG_PATHWAY	Alanine, aspartate and glutamate metabolism	RT		7	0.5	1.4E-2	3.3E-1
KEGG_PATHWAY	Renal cell carcinoma	RT		11	0.8	1.9E-2	3.7E-1
KEGG_PATHWAY	Glycine, serine and threonine metabolism	RT		7	0.5	1.9E-2	3.3E-1
KEGG_PATHWAY	Cysteine and methionine metabolism	RT		7	0.5	2.2E-2	3.4E-1
KEGG_PATHWAY	Nicotinate and nicotinamide metabolism	RT		6	0.4	2.4E-2	3.4E-1
KEGG_PATHWAY	Adipocytokine signaling pathway	RT		10	0.7	3.6E-2	4.3E-1
KEGG_PATHWAY	PPAR signaling pathway	RT		11	0.8	4.0E-2	4.4E-1
KEGG_PATHWAY	Prostate cancer	RT		12	0.8	4.0E-2	4.2E-1
KEGG_PATHWAY	Oocyte meiosis	RT		14	1.0	4.6E-2	4.4E-1

The BMAL1 putative targets in the three most significant pathways are shown in the Figure S5.
