# Supplementary material for: Genome-Wide and Phase-Specific DNA-Binding Rhythms of BMAL1 Control Circadian Output Functions in Mouse Liver
Source: PLoS Biol. 2011 Feb 22;9(2):e1000595. doi: 10.1371/journal.pbio.1000595 (PMC3043000; doi:10.1371/journal.pbio.1000595)
Supplement: Table S5 — PSWM for the E1-E2 motif. E1 goes from position 1 to 13, position 14 corresponds to the spacer, and E2 goes from position 15 to 27. (0.05 MB PDF) [file pbio.1000595.s013.pdf]

Table S5. Position weight matrix for the E1-E2 motif.

	A	C	G	T
**1**	0.21	0.34	0.31	0.15
**2**	0.29	0.20	0.33	0.18
**3**	0.19	0.30	0.40	0.11
**4**	0.22	0.06	0.34	0.39
**5**	0.01	0.99	0.00	0.00
**6**	0.98	0.00	0.00	0.02
**7**	0.01	0.88	0.09	0.03
**8**	0.29	0.07	0.61	0.03
**9**	0.06	0.10	0.08	0.75
**10**	0.04	0.00	0.85	0.11
**11**	0.07	0.29	0.43	0.21
**12**	0.27	0.41	0.17	0.15
**13**	0.22	0.37	0.21	0.20
**14**	0.23	0.30	0.32	0.15
**15**	0.19	0.18	0.54	0.09
**16**	0.16	0.18	0.53	0.14
**17**	0.21	0.32	0.43	0.03
**18**	0.18	0.71	0.09	0.01
**19**	0.62	0.05	0.17	0.16
**20**	0.03	0.47	0.31	0.20
**21**	0.15	0.16	0.65	0.05
**22**	0.12	0.08	0.21	0.60
**23**	0.01	0.03	0.92	0.04
**24**	0.23	0.49	0.19	0.09
**25**	0.29	0.27	0.22	0.23
**26**	0.19	0.26	0.41	0.13
**27**	0.17	0.32	0.38	0.12

E1 goes from position 1 to 13, position 14 corresponds to the spacer and E2 goes from position 15 to 27.
