# Supplementary material for: Genome-Wide and Phase-Specific DNA-Binding Rhythms of BMAL1 Control Circadian Output Functions in Mouse Liver
Source: PLoS Biol. 2011 Feb 22;9(2):e1000595. doi: 10.1371/journal.pbio.1000595 (PMC3043000; doi:10.1371/journal.pbio.1000595)
Supplement: Table S6 — TaqMan probes for ChIP-PCR measurements. (0.04 MB PDF) [file pbio.1000595.s014.pdf]

Table S6. Taqman probes for ChIP-PCR measurements

**Name**	**Sequence 5’->3’**
DbpI2 fwd	TGGGACGCCTGGGTACAC
DbpI2 rev	GGGAATGTGCAGCACTGGTT
DbpI2 probe	FAM-CCCTGATCCGACCTCCAGGCAA-TAMRA
Per1 fwd	AGCCCTCTCAGCCTATGAGAAAGT
Per1 rev	CCCGCCCTGCCTAAATCA
Per1 probe	FAM-TTTAGGGCAGGGCTGGCATTTGCG-TAMRA
Gapdh fwd	CATGGCCTTCCGTGTTCCTA
Gapdh rev	CCTGCTTCACCACCTTCTTGA
Gapdh probe	FAM-CCGCCTGGAGAAACCTGCCAAGTATG-TAMRA
