# Supplementary material for: Genome-Wide and Phase-Specific DNA-Binding Rhythms of BMAL1 Control Circadian Output Functions in Mouse Liver
Source: PLoS Biol. 2011 Feb 22;9(2):e1000595. doi: 10.1371/journal.pbio.1000595 (PMC3043000; doi:10.1371/journal.pbio.1000595)
Supplement: Table S7 — TaqMan probes for mRNA measurements. (0.05 MB PDF) [file pbio.1000595.s015.pdf]

Table S7. Taqman probes for mRNA measurements.

Probe	Sequence 5′ -> 3′
Dbp mRNA fwd	AAGAAGGCAAGGAAAGTCCA
Dbp mRNA rev	TGTACCTCCGGCTCCAGTA
Dbp mRNA probe	FAM-CTCATCCTTCTGTTCCTCAGGCACC-TAMRA
Gys2 mRNA fwd	CATGACCGAGAAGCTAACGA
Gys2 mRNA rev	ATGACGTGTTTACCGTCTGC
Gys2 mRNA probe	FAM-TCTGTCACCTCCTTTAAGAACCAGGCA-TAMRA
Gys2 pre fwd	AGGAACCGAACCATTCAGAC
Gys2 pre rev	CACTGTCCCTCACATCTGCT
Gys2 pre probe	FAM-CTCCGCCGACACTTCAGGCC-TAMRA
March8 mRNA fwd	GATCTCTGCCATCCCTTCTC
March8 mRNA rev	GTTGCTTGGATGACTCATGG
March8 mRNA probe	FAM-TGTCCCAAAGTCTTCTCATTCTGCTCC-TAMRA
March8 pre fwd	TTTCTGCCTCAGCTTTCCTT
March8 pre rev	ACTGGGTTCCCTTCTTTCCT
March8 pre probe	FAM-CACATGACTGGGCGCCAACA-TAMRA
Cry1 mRNA fwd	CTGGCGTGGAAGTCATCGT
Cry1 mRNA rev	CTGTCCGCCATTGAGTTCTATG
Cry1 mRNA probe	FAM-CGCATTTCACATACACTGTATGACCTGGACA-TAMRA
Cry1 pre fwd	CTTCAACCACGCCTAAGACA
Cry1 pre rev	GGAGCTTGTTTCCATCCAAT
Cry1 pre probe	FAM-TCACTCCCATTTACCCAACCCATTG-TAMRA
Qdpr mRNA fwd	AGCTCCTGGACACCCTTAGA
Qdpr mRNA rev	TTAGGCTTCCTGAGTTTGGC
Qdpr mRNA probe	FAM-TCCAGTCATGGAAGGTTTCCACCA-TAMRA
Qdpr pre fwd	CCCAGTCTCCCAAGGATCTA
Qdpr pre rev	GTTAGCAGAAGCAGCACAGC
Qdpr pre probe	FAM-TCCCAGGAGATCCTTTGCCCA-TAMRA
Cry2 mRNA probe	FAM-CCCGGACTACAAACAGACGCGA-TAMRA
Cry2 mRNA fwd	ACCGATGGAGGTTCCTACTG
Cry2 mRNA rev	AGCCTTGGGAACACATCAG
Cry2 pre fwd	GCCTGGCTTGCTATAGTTCC
Cry2 pre rev	ATTCAGTTGCCTTGCCCTAC
Cry2 pre probe	FAM-TTTGGCCACTGCAGCCAAGC-TAMRA
Per1 mRNA fwd	ACCAGCGTGTCATGATGACATAC
Per1 mRNA rev	CTCTCCCGGTCTTGCTTCAG
Per1 mRNA probe	FAM-CCGTCCAGGGATGCAGCCTCTG-TAMRA
Per1 pre fwd	TGTCTTCCTCCCTCCAATTC
Per1 pre rev	GTGGCCAACAGCAAGAACTA
Per1 pre probe	FAM-ACATCACGACCGGCACGAGG-TAMRA
Per2 mRNA fwd	ATGCTCGCCATCCACAAGA
Per2 mRNA rev	GCGGAATCGAATGGGAGAAT
Per2 mRNA probe	FAM-ATCCTACAGGCCGGTGGACAGCC-TAMRA
Per2 pre fwd	CGCACACATTGTAAGATCCC
Per2 pre rev	TTTGCTGGGTTGTGACTAGC

Per2 pre probe	FAM-ACCAGCCGGCAAGAACAGCC-TAMRA
Nr1d1 mRNA fwd	GTGACCTTTCTCAGCACGAC
Nr1d1 mRNA rev	GTTGAACAACGATGCAAAGC
Nr1d1 mRNA probe	FAM-AGCACCTCAAAGGTGCCAGCC-TAMRA
Nr1d1 pre fwd	GGTTGCCCTGCCTGGTTTA
Nr1d1 pre rev	TGCCACCGAGTCGACAGAA
Nr1d1 pre probe	FAM-CACATGTCTTGCTCACCCACTGACACA-TAMRA
Rorc mRNA fwd	AGTCCTTCCGAGAGACATGC
Rorc mRNA rev	TCCCACATTGACTTCCTCTG
Rorc mRNA probe	FAM-CCTTCTACGGCAGCGCACCA-TAMRA
Rorc pre fwd	GCTAATGGGCTTTCTGGAAC
Rorc pre rev	GCATGCAGTCAGTATTTGGG
Rorc pre probe	FAM-CTGGCCCTAGGCACCCTGGA-TAMRA
Nfil3 mRNA fwd	TGACCTGGAGAAACATGGAA
Nfil3 mRNA rev	CGATTTGAGGGACCAATCTT
Nfil3 mRNA probe	FAM-ATGGCCCATTCCTCCCTCCC-TAMRA
Nfil3 pre fwd	AAGTAGGGCAATCTCAGCGT
Nfil3 pre rev	AAGACACACATTTGGCAAGC
Nfil3 pre probe	FAM-TCGGTCGTCACTTTCCGGCTT-TAMRA
Nr1d2 mRNA fwd	AGTAGGTGGATGTTCTCAGACTGAGA
Nr1d2 mRNA rev	ATGGAGACTTGCTCATAGGACACAC
Nr1d2 mRNA probe	FAM-CAGAAATAGTTACCTGTGCAACACTGGAGGGAG-TAMRA
Nr1d2 pre fwd	TCAGTTCTTTCTTGGGCCTT
Nr1d2 pre rev	AACCTAAACCACGGAAATGC
Nr1d2 pre probe	FAM-TGCCATCCCAGAGGGCAACA-TAMRA
Tef mRNA fwd	GCCGAGCTTCGCAAGGA
Tef mRNA rev	ACAGGTTACAAGGGCCCGTACT
Tef mRNA probe	FAM-ACACGATGGTCTTGCACTTGCCCA-TAMRA
Tef pre fwd	TGGGAATCACAATAGCAGGA
Tef pre rev	CAAATACCCAGGCCAAGTCT
Tef pre probe	FAM-CGGGCTTCATCGAATTGCCC-TAMRA
Bhlhb3 mRNA fwd	GCGAGACGATACCAAGGATAC
Bhlhb3 mRNA rev	TCAGATGTTCGGGCAGTAAA
Bhlhb3 mRNA probe	FAM-CCGAATTAATGAATGCATTGCTCAGC-TAMRA
Bhlhb3 pre fwd	GAAACTCTGCCCGCAAATA
Bhlhb3 pre rev	GGAGTATCCAGCAAGCCACT
Bhlhb3 pre probe	FAM-CCAGCCCTGAGCTTTCCTAACTGTG-TAMRA
