# Supplementary material for: Genome-Wide and Phase-Specific DNA-Binding Rhythms of BMAL1 Control Circadian Output Functions in Mouse Liver
Source: PLoS Biol. 2011 Feb 22;9(2):e1000595. doi: 10.1371/journal.pbio.1000595 (PMC3043000; doi:10.1371/journal.pbio.1000595)
Supplement: Table S8 — Annealing primers for EMSA. (0.04 MB PDF) [file pbio.1000595.s016.pdf]

Table S8: Annealing primers for EMSA.

Name	Sequence 5'->3'
Per2 fwd	CTAGCCGGTCACGTTTTCCACTATGTGACAC
Per2 rev	TCGAGTGTCACATAGTGGAAAACGTGACCGG
Dbp-P fwd	CTAGCATAGCACGCGCAAAGCCATGTGCTTC
Dbp-P rev	TCGAGAAGCACATGGCTTTGCGCGTGCTATG
Dbp-I2 fwd	CTAGCGACTCACGTGGCGAGGGAATGTGCAGC
Dbp-I2 rev	TCGAGCTGCACATTCCCTCGCCACGTGAGTCG
sp6 fwd	CTAGCTGCTCACGTGTCGCGAAACGTGACGC
sp6 rev	TCGAGCGTCACGTTTCGCGACACGTGAGCAG
sp7 fwd	CTAGCTGCTCACGTGTCTGCGAAACGTGACGC
sp7 rev	TCGAGCGTCACGTTTCGCAGACACGTGAGCAG
sp8 fwd	CTAGCTGCTCACGTGTCTGTCGAAACGTGACGC
sp8 rev	TCGAGCGTCACGTTTCGACAGACACGTGAGCAG
sp9 fwd	CTAGCTGCTCACGTGTCTGCTCGAAACGTGACGC
sp9 rev	TCGAGCGTCACGTTTCGAGCAGACACGTGAGCAG
mE1-E2 fwd	CTAGCTGCTACCGGTTCTGCGAAACGTGACGC
mE1-E2 rev	TCGAGCGTCACGTTTCGCAGAACCGGTAGCAG
E1-mE2 fwd	CTAGCTGCTCACGTGTCTGCGATGGAACACGC
E1-mE2 rev	TCGAGCGTGTTCCATCGCAGACACGTGAGCAG
mE1-mE2 fwd	CTAGCTGCTACCGGTTCTGCGATGGAACACGC
mE1-mE2 rev	GACGATGGCCAAGACGCTACCTTGTGCGAGCT
sp7 2D gel fwd	CTAGCTGC
sp7 2D gel rev	CGTCACGTTTCGCAGACACGTGAGCAGCTAG
