# Supplementary material for: Genome-Wide and Phase-Specific DNA-Binding Rhythms of BMAL1 Control Circadian Output Functions in Mouse Liver
Source: PLoS Biol. 2011 Feb 22;9(2):e1000595. doi: 10.1371/journal.pbio.1000595 (PMC3043000; doi:10.1371/journal.pbio.1000595)
Supplement: Table S9 — Annealing primers for transactivation assays. (0.05 MB PDF) [file pbio.1000595.s017.pdf]

Table S9: Annealing primers for transactivation assays.

Name	Sequence 5'->3'
Dbp-I2 WT Fwd	GTACCACTCACGTGGCGAGGGAATGTGCAACTC
Dbp-I2 WT Rev	TCGAGAGTTGCACATTCCCTCGCCACGTGAGTG
Dbp-I2 E1-mE2 Fwd	GTACCACTCACGTGGCGAGGGTGGAATCAACTC
Dbp-I2 E1-mE2 Rev	TCGAGAGTTGATTCCACCCTCGCCACGTGAGTG
Dbp-I2 sp10 Fwd	GTACCACTCACGTGGCGACTAGGGAATGTGCAC
Dbp-I2 sp10 Rev	TCGAGTGCACATTCCCTAGTCGCCACGTGAGTG
Per2 WT Fwd	GTACCGGTCACGTTTTCCACTATGTGACTAGCC
Per2 WT Rev	TCGAGGCTAGTCACATAGTGGAAAACGTGACCG
Per2 E1-mE2 Fwd	GTACCGGTCACGTTTTCCACTGGATTACTAGCC
Per2 E1-mE2 Rev	TCGAGGCTAGTAATCCAGTGGAAAACGTGACCG
Per2 sp10 Fwd	GTACCGGTCACGTTTTCCTAGCACTATGTGACC
Per2 sp10 Rev	TCGAGGTCACATAGTGCTAGGAAAACGTGACCG
Dbp-I2 mE1-E2 Fwd	GTACCACTACCTGGGCGAGGGAATGTGCAACTC
Dbp-I2 mE1-E2 Rev	TCGAGAGTTGCACATTCCCTCGCCCAGGTAGTG
Dbp-I2 mE1-mE2 Fwd	GTACCACTACCTGGGCGAGGGTGGAATCAACTC
Dbp-I2 mE1-mE2 Rev	TCGAGAGTTGATTCCACCCTCGCCCAGGTAGTG
Dbp-I2 E1-E1 Fwd	GTACCACTCACGTGGCGAGGGCACGTGCAACTC
Dbp-I2 E1-E1 Rev	TCGAGAGTTGCACGTGCCCTCGCCACGTGAGTG
Dbp-I2 E2-E2 Fwd	GTACCACTAATGTGGCGAGGGAATGTGCAACTC
Dbp-I2 E2-E2 Rev	TCGAGAGTTGCACATTCCCTCGCCACATTAGTG
Dbp-I2sp20 Fwd	GTACCACTCACGTGGCGAGGGGCGAGGGCGAGGGAATGTGCAC
Dbp-I2 sp20 Rev	TCGAGTGCACATTCCCTCGCCCTCGCCCCTCGCCACGTGAGTG
Dbp-I2 sp17 Fwd	GTACCACTCACGTGGCGAGGGAGGGCGAGGGAATGTGCAGCGC
Dbp-I2 sp17 Rev	TCGAGCGCTGCACATTCCCTCGCCCTCCCTCGCCACGTGAGTG
Dbp-I2 sp10 Fwd	GTACCACTCACGTGGCGAGGGGCGAATGTGCAAGGGAGGGCGC
Dbp-I2 sp10 Rev	TCGAGCGCCCTCCCTCGTGCACATTCCCCTCGCCACGTGAGTG
Dbp-I2 sp8 Fwd	GTACCACTCACGTGGCGAGGGGAATGTGCACGAGGGAGGGCGC
Dbp-I2 sp8 Rev	TCGAGCGCCCTCCCTCGTGCACATTCCCCTCGCCACGTGAGTG
Dbp-I2 sp7 Fwd	GTACCACTCACGTGGCGAGGGAATGTGCAGCGAGGGAGGGCGC
Dbp-I2 sp7 Rev	TCGAGCGCCCTCCCTCGCTGCACATTCCCTCGCCACGTGAGTG
Dbp-I2 sp6 Fwd	GTACCACTCACGTGGCGAGGAATGTGCAGGCGAGGGAGGGCGC
Dbp-I2 sp6 Rev	TCGAGCGCCCTCCCTCGCCTGCACATTCCTCGCCACGTGAGTG
Dbp-I2 sp4 Fwd	GTACCACTCACGTGGCGAAATGTGCAGGGGCGAGGGAGGGCGC
Dbp-I2 sp4 Rev	TCGAGCGCCCTCCCTCGCCCCTGCACATTTCGCCACGTGAGTG
