# Supplementary material for: Genome-Wide and Phase-Specific DNA-Binding Rhythms of BMAL1 Control Circadian Output Functions in Mouse Liver
Source: PLoS Biol. 2011 Feb 22;9(2):e1000595. doi: 10.1371/journal.pbio.1000595 (PMC3043000; doi:10.1371/journal.pbio.1000595)
Supplement: Text S1 — Supplementary methods. (0.09 MB PDF) [file pbio.1000595.s018.pdf]

Supplementary Methods

1 Deconvolution method for ChIP-Seq data

We write the two counts distributions as convolutions with a fixed kernel $\mathcal{M}$:

$$\mathrm{P}^{\pm}_{\mathrm{tag}}(n) \;=\; \sum_{k>n-\ell} \mathcal{M}\big(\pm(k-n)\big)\mathrm{P}_{\mathrm{bind}}(k)\;. \tag{1}$$

We use quadratic programming with linear constraints to find the optimal solution. To derive the kernel, we assume that every position in a chromosome (or genome) of length L is equally likely to break and that the chromosome is fragmented into Lq pieces (of average size $1/q$). If all breaking events are independent, then the length distribution is geometric with parameter q.

$$\mathrm{P}^{(0)}_{\mathrm{len}}(\lambda) \;=\; q(1-q)^{\lambda-1}\;.$$

To reduce the number of parameters and to avoid the complication of boundary effects, we set $L = \infty$. The experimental protocols typically involve selecting a band in a gel, namely using only fragments with length in the range $[\mu, \nu]$. In the sequel we set $\nu = \infty$ since this is an exponentially small perturbation. However the lower bound is important and the effective length distribution is

$$\mathrm{P}_{\mathrm{len}}(\lambda) \;=\; \begin{cases} 0 & \text{if } \lambda \;<\; \mu\;, \\ q(1-q)^{\lambda-\mu} & \text{if } \lambda \;\geq\; \mu\;. \end{cases}$$

The fraction of transcription factors bound at position k in the genome is $\mathrm{P}_{\mathrm{bind}}(k)$ and we compute the probability $\mathrm{P}^{-}_{\mathrm{base}}(b)$ that a sonication fragment overlapping k breaks at $b \geq k$, thereby producing a count at position b on the negative strand. Such a fragment can start at any position $a \leq k$ having length $\lambda = b - a + 1$. The probability of such an fragment is therefore:

$$\begin{aligned} &\mathrm{P}^{-}_{\mathrm{base}}(b) \\ &\quad=\; C^{-1}\sum_{k\leq b}\sum_{a\leq k}\mathrm{P}_{\mathrm{len}}(b-a+1)\mathrm{P}_{\mathrm{bind}}(k) \\ &\quad=\; \sum_{k\leq b}\mathcal{K}(b-k)\mathrm{P}_{\mathrm{bind}}(k)\;, \end{aligned}$$

where

$$\mathcal{K}(n) \;=\; C^{-1}\begin{cases} 1 & \text{if } 0 \;\leq\; n \;<\; \mu\;, \\ (1-q)^{n-\mu} & \text{if } n \;\geq\; \mu\;, \end{cases}$$

and C is a normalization constant to ensure $\sum_n \mathcal{K}(n) = 1$.

If we now take into account that tags have a length $\ell < \mu/2$ (typically $\ell = 38$bp for standard Solexa GAII) and therefore cover ℓ positions downstream of the break point, the distribution is affected as follows:

$$\begin{aligned} & \mathrm{P}^+_{\mathrm{tag}}(n) \\ &= C^{-1} \sum_{a=n-\ell+1}^{n} \mathrm{P}^+_{\mathrm{base}}(a) \\ &= \sum_{k>n-\ell} \mathcal{M}(k-n)\mathrm{P}_{\mathrm{bind}}(k) \ , \end{aligned} \tag{2}$$

with

$$\mathcal{M}(n) = \\ C^{-1} \begin{cases} (\ell+n)q & \text{if } 1-\ell \leq n < 0 \ , \\ \ell q & \text{if } 0 \leq n \leq \mu-\ell \ , \\ q(\mu-n)+1-(1-q)^{n+\ell-\mu} & \text{if } \mu-\ell < n < \mu \ , \\ (1-q)^{n-\mu}(1-(1-q)^{\ell}) & \text{if } n \geq \mu \ . \end{cases}$$

We next propose an algorithm for de-convoluting Eq. 2 for both strands simultaneously, meaning we solve the following matrix equation for $\mathrm{P}_{\mathrm{bind}}$

$$\begin{pmatrix} \mathrm{P}^+_{\mathrm{tag}} \\ \mathrm{P}^-_{\mathrm{tag}} \end{pmatrix} = \begin{pmatrix} \mathcal{M}^T \\ \mathcal{M} \end{pmatrix} \cdot \mathrm{P}_{\mathrm{bind}} \ ,$$

with the constraints

$$\sum_k \mathrm{P}_{\mathrm{bind}}(k) = 1 \ , \ \text{and } \mathrm{P}_{\mathrm{bind}}(k) \geq 0 \ \forall k \ .$$

Here we represented the convolution kernel as a matrix $\mathcal{M}_{ij} = \mathcal{M}(i-j)$. We solve this problem by searching for the vector $\mathrm{P}_{\mathrm{bind}}$ which minimizes the error

$$\mathcal{E}^2_0(\mathrm{P}_{\mathrm{bind}}; \mu, q, \ell) = \left\| \mathrm{P}^+_{\mathrm{tag}} - \mathcal{M}^T \mathrm{P}_{\mathrm{bind}} \right\|^2 + \left\| \mathrm{P}^-_{\mathrm{tag}} - \mathcal{M} \mathrm{P}_{\mathrm{bind}} \right\|^2 \ .$$

We use quadratic programming to find such a minimizer satisfying the constraints. This is applied within each enriched region detected *a priori* by MACS [1].

2 Example

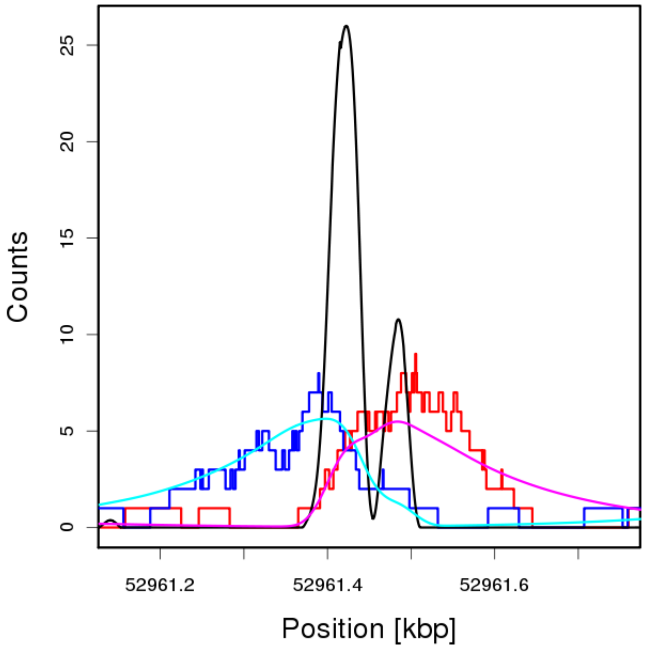

Figure 1: Deconvolution algorithm applied to the promoter site of Dbp. Black curve is P_{bind}, smooth curves are the theoretical signal for the positive and negative strands Eq. 1 and rough curves are the raw data.

References

[1] Zhang Y, Liu T, Meyer C, Eeckhoute J, Johnson D, et al. (2008) Model-based analysis of chip-seq (macs). Genome Biol 9: R137.
